# Relevance of Circulating Tumor Cells as Predictive Markers for Cancer Incidence and Relapse

**DOI:** 10.3390/ph15010075

**Published:** 2022-01-06

**Authors:** Chaithanya Chelakkot, Hobin Yang, Young Kee Shin

**Affiliations:** 1 Bio-MAX/N-Bio, Bio-MAX Institute, Seoul National University, Seoul 08226, Korea; cgchaithanyalakshmi@gmail.com; 2 Genobio Corp., Seoul 08394, Korea; 3 Research Institute of Pharmaceutical Science, Department of Pharmacy, College of Pharmacy, Seoul National University, Seoul 08226, Korea; dbdyd99@naver.com; 4 Department of Molecular Medicine and Biopharmaceutical Sciences, Graduate School of Convergence Science and Technology, Seoul National University, Seoul 08226, Korea

**Keywords:** liquid biopsy, circulating tumor cells, predictive biomarker, companion diagnostics, precision medicine

## Abstract

Shedding of cancer cells from the primary site or undetectable bone marrow region into the circulatory system, resulting in clinically overt metastasis or dissemination, is the hallmark of unfavorable invasive cancers. The shed cells remain in circulation until they extravasate to form a secondary metastatic lesion or undergo anoikis. The circulating tumor cells (CTCs) found as single cells or clusters carry a plethora of information, are acknowledged as potential biomarkers for predicting cancer prognosis and cancer progression, and are supposed to play key roles in determining tailored therapies for advanced diseases. With the advent of novel technologies that allow the precise isolation of CTCs, more and more clinical trials are focusing on the prognostic and predictive potential of CTCs. In this review, we summarize the role of CTCs as a predictive marker for cancer incidence, relapse, and response to therapy.

## 1. Introduction

Tissue biopsy is the gold standard for cancer diagnosis and profiling [1]; however, determining a treatment regimen for advanced diseases based solely on the information available from tissue biopsies is challenging [2]. Circulating tumor cells (CTCs) were first identified almost a century ago by the Australian physician Thomas Ashworth in the blood of metastatic cancer patients [3]. However, it was not until the past two decades that their prognostic potential was elucidated, primarily because of the significant technical challenges in isolating these rare cells from an overwhelming background of peripheral blood mononuclear cells (PBMCs). Liquid biopsy (LBx) refers to isolating cancer cells or cancer cell-derived components from body fluids for further analysis [4]. This approach is gaining significant attention due to its noninvasive nature and minimal risk associated with longitudinal sampling [5,6]. The clinical validity of CTC enumeration was supported by a series of multicenter studies which led to the inclusion of CTCs for cancer staging for breast cancer (BC) patients (cM0; no clinical or radiologic evidence of distant metastases, but deposits of molecularly or microscopically detected tumors in the circulating blood, bone marrow, or other non-regional nodal tissue that are no larger than 0.2 mm in a patient without symptoms or signs of metastases) by the 8th edition of the American Joint Committee on Cancer (AJCC) cancer staging manual and in the World Health Organization’s WHO Classification of Tumours of the Breast [7,8]. With the recent introduction of a multitude of remarkable technologies that isolate CTCs from peripheral blood (PB) with high sensitivity and specificity [9], the bottleneck in the CTC research field has shifted from their efficient isolation to their molecular analysis and demonstration of their clinical utility as potential prognostic, predictive, and pharmacodynamic biomarkers.

## 2. Enrichment and Isolation of CTCs

Enrichment of CTCs from PB is a herculean task as these cells are scarce, ranging from 1–10 cells among five million white blood cells (WBCs). Several techniques for CTC isolation have been made known with seminal discoveries made in recent years [9]. These techniques exploit either the physical or biological properties of CTCs. Among the physical properties of CTCs, cell size, density, or deformability are exploited for CTC isolation. On the other hand, approaches that utilize biological properties primarily rely on the cell surface markers specifically expressed on cancer cells (positive selection) or leukocytes (negative selection); negative selection relies on leukocyte depletion using leukocyte-specific markers such as CD45 [10]. Strategies combining both positive and negative selection and size-based label-free techniques for better enrichment have also been demonstrated [11]. Table 1 summarizes the devices and techniques for CTC isolation [12,13,14,15,16,17,18,19,20,21,22,23,24,25,26,27,28,29,30,31,32,33,34,35,36,37,38,39,40,41,42,43,44,45].

An extensive review of the methods for CTC isolation techniques and strategies is beyond the scope of this paper and has been elegantly reviewed elsewhere [9,46,47]. A few classic methods are briefly mentioned below.

Enrichment of CTCs using the positive selection method is the most widely used strategy, and the only Food and Drug Administration (FDA)-approved CTC enrichment system, the CellSearch^®^ system from Menarini silicon biosystems, follows this strategy. This method, which is the “gold standard for LBx,” uses anti-epithelial cell adhesion molecule (EpCAM) antibody (AB)-conjugated ferrofluid nanoparticles to separate CTCs under a magnetic field. For CTC detection, the enriched cells are immuno-stained for epithelial marker cytokeratins (CKs) 8, 18, and 19 along with the leukocyte marker CD45. Mononuclear cells (as detected by 4′, 6-diamidino-2-phenylindole (DAPI) staining) positive for CKs and negative for CD45 are classified as CTCs. Reproducibility and reliability of CTCs isolated by the CellSearch^®^ system have been demonstrated in a series of hallmark studies, which subsequently led to its FDA approval for clinical diagnosis [48,49,50,51,52] for BC, prostate cancer (PC), and colorectal cancer (CRC).

In addition to EpCAM, numerous other cancer-specific proteins are used for CTC isolation. These approaches rely on the fact that cancer cells undergoing epithelial-mesenchymal transition (EMT) have reduced expression of epithelial markers and show elevated expression of mesenchymal/metastatic markers such as VIM, EGFR, MUC1, HER2, and MET [53]. In recent years, numerous systems that rely on the AB-based method for CTC isolation using these biomarkers for a multivalent capture of CTCs have been developed [22,54,55]. A more recent strategy for CTC isolation combines the immune-magnetophoretic approach with a microfluidic approach for more efficient CTC capture [17,56,57,58].

Advances in microfabrication have resulted in the development of microstructures that enable cell capture [59,60,61,62]. Microfluidic chips with multiple arrays of microscopic posts coated with cancer-specific ABs (e.g., anti-EpCAM AB) increase the interactive surface between the putative CTCs in the bloodstream and the ABs, resulting in increased enrichment efficacy. The captured cells can then be detected within the microfluidic chips by immunostaining or released from the chips using a suitable buffer for further molecular analysis. The challenge here is to overcome the significant drop in the affinity of an AB once they are immobilized on the surface due to the denaturation of their three-dimensional structure. The laminar flow within the microfluidic chips also poses a significant hindrance to the efficient interaction between the CTCs in the bloodstream and the immobilized ABs within the chips. To resolve this issue, Sequist et al. designed a silicon chip with about 78,000 anti-EpCAM AB-coated micro-posts, which are spatially arranged in the flow path to counter the drag force in the laminar flow, achieving a 10^6^ fold enrichment of CTCs [23,63].

AB bias, capturing only certain subtypes of CTCs, is a major drawback associated with positive enrichment of CTCs, which could be overcome using a label-free CTC enrichment strategy [20,31,32,33,36,37,38,64,65]. In a label-free method, the larger size of CTCs (diameter of approximately 30 µm compared to 6–8 µm of surrounding leukocytes) is exploited for the size-based enrichment of CTCs using a filtration or inertial flow method. In the widely used ISET^®^ (isolation by size of tumor cells/trophoblastic cells) system and other filtration-based technology, a specially designed filter is employed [32,33]. As the blood sample percolates through these specialized filters, larger cells (presumably including CTCs) are collected, which can then be used for molecular and genomic analysis [66,67]. However, clogging of the filtration units due to the large CTC clusters or other tissues in the blood sample results in very high background cell contamination and often requires a second purification step for high-purity CTC yield.

Hydrodynamic microfluidic methods allow size-based positive enrichment of CTCs and rely on the inertial drag forces and lift forces within the microfluidic chamber for sorting larger cells from smaller cells. CTCs can be separated from blood cells using the differences in inertial life force and Dean drag force between them [68,69,70]. Spiral microchannels for CTC separation are used mainly in the microfluidic separation of CTCs and are proving to be a powerful tool to achieve efficient cell sorting [71]. CTCs, being larger, incline toward the inner wall side and can be dragged into separate collection tubes with minimal contamination from smaller white blood cells (WBCs), which are dragged towards the outer wall within the microfluidic chamber. Spiral inertial microfluidic channels were shown to prevent contamination with non-target cells, a major challenge in label-free CTC isolation. A study by Nivedita et al. reported the removal of more than 90% of non-target cells while maintaining the viability of CTCs [72]. Deterministic lateral displacement allowing the preferential migration of cells depending on the hydrodynamic forces is another method that allows CTC separation in a label-free manner [73,74]. Dielectrophoretic separation of cells based on the differences in the electric charge carried by the cells has proven to be a promising approach in CTC capture. The DEPArray^TM^ device employs this strategy in single-cell isolation of CTCs, after an initial upstream separation either using a labeled or label-free method [75,76].

The lack of FDA-approved CTC isolation devices other than the CellSearch^®^ system has prompted several attempts to develop an efficient, high-throughput, user-friendly, low-cost device that is easily adaptable for mass manufacture and suitable for routine clinical use. An impressive array of novel techniques is now available in the market, each claiming high-efficiency CTC capture compared to the CellSearch^®^ system, shifting the rate-limiting step in CTC research from enrichment to their characterization and demonstration of clinical utility. Studies are now focusing on the characterization of CTCs at the molecular and genomic level and utilizing the information to develop a personalized treatment regimen. CTC-based companion diagnostics (CDx) development has now taken center stage in CTC research. Recently the FDA approved several PCR- or next-generation sequencing (NGS)-based mutation tests in cancers for CDx. The development of CTC-based CDx platforms is expected to transform the practice of precision medicine and open new era technologies for cancer detection, disease monitoring, and treatment planning.

## 3. Clinical Utility of CTCs as a Biomarker in Solid Tumors

The CTC count and their molecular and genomic signature are heterogenic and subjected to dynamic changes within a patient in response to disease progression or therapy. The lifespan of CTCs in the bloodstream is also very short, approximately twenty-four hours, and the CTC repertoire is continuously replenished from the primary tumor or metastatic sites and dormant cells in the bone marrow. CTCs can thus be prevailing in the bloodstream when conventional methods fail to detect the presence of the disease. CTCs thereby can be considered a monitoring biomarker, a tool to monitor the disease in real time and predict recurrence or relapse in the non-metastatic phase.

The clinical utility of CTCs in the non-metastatic phase can be assessed by monitoring the CTCs longitudinally for evaluating disease progression during therapy and correlating it with target tumor lesion, similar to studies on conventional radiological outcomes. CTC detection in non-metastatic BC has been an independent biomarker for predicting metastatic relapse [77,78]. When the REMAGUS-02 trial evaluated 115 non-metastatic patients with large operable or locally advanced BC receiving neoadjuvant therapy, a positive CTC result before chemotherapy was an independent prognostic factor for distant-metastasis-free survival (DMFS) and overall survival (OS) in BC [79]. Late recurrence accounts for 50% of the recurrences in hormone receptor (HR)-positive (HR^+^) BC patients with no specific biomarker to predict the relapse. A recent study by Sparano et al. on HR^+^ BC patients, five years after initial diagnosis, showed that a positive CTC result could be an independent prognostic factor for late clinical recurrence [80]. A secondary analysis of the randomized PERSEVERE trial (BRE12-158 study) evaluated the association between residual disease in triple-negative breast cancer (TNBC) patients post neo-adjuvant therapy and disease recurrence. The presence of a single CTC and one ctDNA alteration significantly reduced the distant disease-free survival (DDFS), compared with the patients who were positive only for one of these markers, a finding that is consistent with the study by Sparano et al. [81]. Combination analysis of ctDNA and CTCs, indicating the presence of minimal residual diseases in TNBC patients after neo-adjuvant chemotherapy and surgery, could thus be an independent marker in risk stratification, addressing an unmet need in selecting patients for intervention treatments.

The CTC count has been shown to be a prognostic biomarker in a series of studies in several different cancer types, including BC, PC, CRC, and other metastatic cancers [5,48,49,50,51,52,82,83,84,85,86,87]. In a pivotal clinical trial using the CellSearch^®^ system, metastatic BC (mBC) patients with a baseline positive CTC count of ≥5 showed a significantly reduced OS rate (10.9 months) compared to patients with <5 CTCs (21.9 months). Similarly, a reduction in CTC count to <5 after the initiation of therapy was an independent biomarker for OS; patients who had a reduction in CTC count from ≥5 to <5 post-treatment had improved OS (10.6 months) compared to patients whose CTC count remained ≥5 at all time points (4.1 months) [48,85]. In metastatic castration-resistant PC (mCRPC), a similar CTC cutoff of 5 at baseline as well as after initiation therapy proved to be an independent biomarker to predict OS. CTC count could predict the OS better than the conventional biomarker prostate-specific antigen (PSA) in mPC patients [52]. In metastatic CRC (mCRC), the CTC count cutoff value was only 3 CTCs; a baseline count of ≥3 CTCs was a significant predictor of OS. Patients who had a CTC count ≥3 at baseline and at all timepoints had poor prognosis, with median OS being 3.9 months compared to 18.6 months in patients whose CTC count remained <3 at all time points [51,87]. In metastatic renal cancer (mRC) patients receiving first-line tyrosine kinase inhibitors (TKI), the presence of 3 or more CTCs at baseline was found to be associated with poor PFS and OS. However, in this cohort, baseline and dynamic CTC counts were not predictive of radiological response [88].

In a pooled analysis of individual patient data from 2436 mBC patients, the prognostic utility of CTCs in the stratification of patients with stage IV cancer was demonstrated. Stage IV patients could be divided into two subgroups of stage IV_indolent_ (<5 CTCs in 7.5 mL blood) and stage IV_aggressive_ (≥5 CTCs in 7.5 mL blood) by CTC count, independent of other clinical and molecular variables for patient stratification in prospective clinical trials [89]. The decrease in CTCs during treatment is an independent prognostic factor in mBC. In the interventional clinical trial SWOG S0500 by Smerage et al., the prognostic role of CTC enumeration was demonstrated, while the study failed to prove that an early switching to an alternate cytotoxic therapy in patients with persistently high CTC count after first-line chemotherapy is beneficial [90]. An ancillary study of the LANDSCAPE trial showed that the presence of CTCs during treatment correlated with brain metastasis and could prove to be a tool to detect early treatment failure in mBC. An absence of CTC decrease at day 21 in patients undergoing a combination therapy of lapatinib and capecitabine in HER2-positive (HER2^+^) mBC patients could be a prognostic biomarker and help select patients for early whole-brain irradiation [91].

In addition to single CTCs, CTC clusters have been shown to be associated with worse clinical outcomes in different cancer types [92,93,94,95,96,97]. Customized microfluidic chips that aid the specific isolation of CTC clusters have been developed [28,61,98,99,100]. Cluster-Chip is a first-generation device of this type and allows biomarker independent isolation of CTC clusters; separation relies on the cell–cell junction interaction within the cluster. The chip has microfabricated bifurcated traps that can efficiently capture even 2-celled clusters from unprocessed whole blood [61]. CTC clusters in large numbers are observed in the pre-operative blood samples collected from the pulmonary vein in early-stage BC and lung cancer (LC) patients [94]. In mBC and mCRPC patients, CTC clusters add value to the prognostic significance of CTCs, and larger CTC-cluster size and persistent presence of CTC clusters is associated with worse outcome in patients [86,101,102,103]. Longitudinal analysis of CTC clusters in mBC patients showed significant correlation between size of the cluster and patient’s OS, adding prognostic value to the CTCs, and significantly increasing the risk of death in these patients [97]. A retrospective study of the SWOG0500 evaluated the prognostic significance of CTC clusters and showed that the presence of CTC doublets and clusters is associated with worse OS compared to patients with only doublets, no doublets, or no clusters. However, the presence of CTC clusters is not an independent factor for predicting mortality in mBC patients starting first-line therapy [104]. A clinical trial is currently investigating the clinical relevance of targeting CTC clusters in cancer treatment (NCT03928210).

A clinically unmet need and challenging application of CTCs is their utilization for early detection of cancers. A few studies have shown that the presence of CTCs could specifically predict the disease in people with a high risk of cancer due to familial predisposition or other comorbidities associated with a high risk of malignant disease [105,106]. CTC screening was shown to be a highly sensitive predictive biomarker for cancer detection, with CTC count being significantly associated with a higher risk of malignant disease. This study also demonstrated that a shift in the diet of patients with CTCs to a highly nutrient-rich diet enriched with anti-carcinogenic properties reduced CTC counts [105]. In patients with borderline PSA levels of 4–10 ng/mL, a CTC-based blood test could correctly identify PC patients with 80% specificity and more than 90% sensitivity [106].

**Figure 1:**
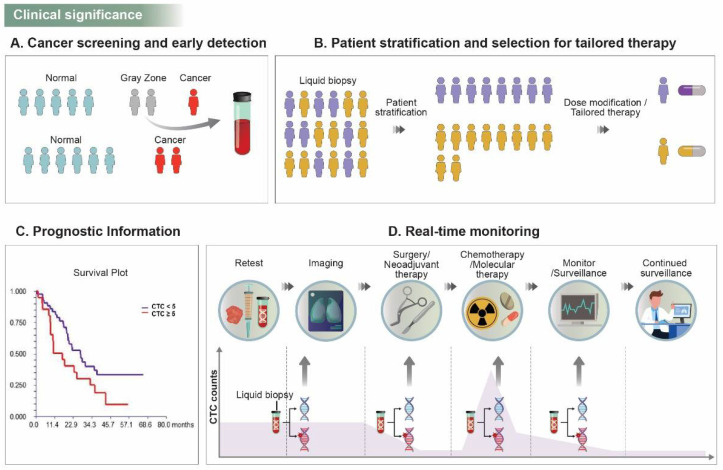
Significance of CTC research in clinical translation. (**A**) CTCs can help in screening and identifying patients who are predisposed to cancer (represented as gray zone) due to familial history, intermediated expression of cancer-associated markers (such as PSA), or other comorbidities, and thereby prevent overtreatment (overdiagnosis) or undertreatment of patients in gray zone. (**B**) Patient stratification based on CTC count or CTC expression of clinically targetable markers can improve patient outcomes by altering the therapeutic strategy and developing more personalized therapy decisions. (**C**) CTC can provide prognostic information on cancer progression or early/late recurrences in different cancer types. (**D**) CTCs allow for real-time monitoring at different stages of cancer treatment, and combined with conventional radiological/histological information can help in treatment decisions while being a tool for continued surveillance in the DFS period.

Several randomized multicenter trials in the past few decades have been designed to prove the potential predictive use of CTCs as treatment-focused biomarkers. As the trial results are reported, CTCs are emerging as a pharmacodynamic/response biomarker, an efficient tool to measure response to therapy, and a predictive biomarker to help determine the risk of relapse and late recurrences after medical treatment. Taken together, CTC analysis is meaningful in clinical translation in terms of early diagnosis, patient stratification, and drug selection, prognostic information, real-time monitoring, and personalized medicine for better patient outcomes (Figure 1).

### 3.1. CTC Enumeration as a Predictive or Pharmacodynamics/Response Biomarker for Response to Therapy

While early-stage solid tumors can be cured by radical surgery, 30–40% patients show relapse/late recurrence. A reliable biomarker to predict relapse/late recurrence due to residual or dormant cancer cells is still not enough [107,108]. Similarly, the prediction of the response to therapy at an early phase of treatment is critical in metastatic cancers [109]. The presence of CTCs in the bloodstream is an indication of residual cancer cells and can be a predictive biomarker for relapse as well as response to medical intervention or therapy [110].

CTCs have been demonstrated to be predictive of the benefit of radiotherapy in early-stage BC patients. CTC-positive patients who received radiotherapy had longer OS compared to patients who did not receive any therapy [111]. A retrospective study by Bidard et al. showed that bevacizumab combined with first-line therapy might modify the predictive value of CTCs during treatment [112]. In another interesting study, it was shown that the baseline CTC count could be a predictive marker to decide which patient group would benefit from more aggressive treatments in mBC patients. HR^+^ mBC patients having a CTC count ≥5, did not benefit from first-line endocrine therapy. The same study also showed that HER2-negative (HER2^−^) patients with a CTC count of ≥5, who would have had a poor prognosis, benefited when chemotherapy was combined with bevacizumab [113]. These studies suggested that the negative prognostic impact of a high CTC count could be reversed, to an extent, with combination therapy with anti-angiogenic agents. The clinical utility of CTC counts in deciding the treatment regimen was elegantly demonstrated in the STIC CTC randomized clinical trial. This study evaluated the predictive potential of CTC count as an alternative to other clinical evaluations to determine the first line of therapy in HR^+^ HER2^−^ mBC patients. The treatment choice, which is either chemotherapy or endocrine therapy, is usually physician-driven. In this randomized trial, the patients were grouped into two arms, where the treatment decision was clinician-driven or was based on a CTC-driven choice. The CTC-driven treatment choice (chemotherapy if CTC count is high, and endocrine therapy if CTC count is low) was shown to be non-inferior to the physician-driven arm, demonstrating for the first time that CTC count may be a reliable biomarker for guiding treatment decision choice between chemotherapy and endocrine therapy in HR^+^ HER2^−^ mBC patients [114].

The predictive role of CTC count has been evaluated in studies of EGFR tyrosine kinase inhibitors (TKIs) in non-small cell LC (NSCLC). A single-arm phase II trial of erlotinib and pertuzumab with relapsed or refractory metastatic NSCLC (mNSCLC) showed that a high baseline CTC count correlated with response to treatment and radiographic response, but not with progression-free survival (PFS) and positron emission tomography (PET) response. The study also showed that a decrease in CTC count following treatment predicted a longer PFS [115]. In a multicenter prospective study on small cell LC (SCLC) to investigate the correlation between change in CTC count and response to chemotherapy, it was shown that absolute CTC count after the first cycle of therapy was a strong predictor for response to chemotherapy and OS [116]. Several studies have reaffirmed these results and showed the reliability of CTC enumeration as a minimally invasive method to predict response to therapy and risk stratification in SCLC patients [117,118].

Heller et al. recently showed the clinical utility of CTC number as a surrogate biomarker for survival in mCRPC compared to the conventionally used clinical biomarker PSA. The study evaluated data from 5 randomized phase-III trials (COU-AA-301, AFFIRM, ELM-PC-5, ELM-PC-4, and COMET-1), and showed that both a negative CTC result at week 13 and a reduction of CTC count from ≥5 at baseline to <4 at week 13 were robust and meaningful predictors for prolonged survival [119]. In the ongoing SWOG S1216 randomized trial, the preliminary results suggest that CTCs could help stratify patients into those who would benefit from hormone therapy and those who would require more aggressive therapy or early therapeutic interventions [120].

### 3.2. Molecular Analysis of CTCs to Identify Their Role as a Predictive Biomarker

**Figure 2:**
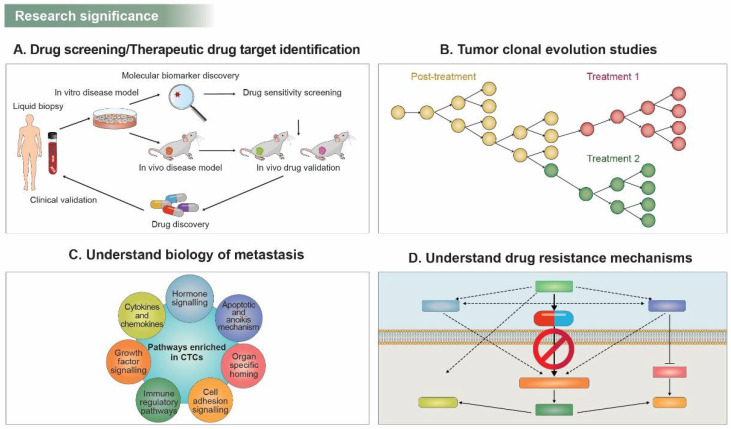
Significance of CTCs in basic research. (**A**) Biomarker discovery through CTC culture and CTC based in vivo models effectively enables drug screening and therapeutic drug target identification. (**B**) Tumor cells undergo genetically distinct subclonal and clonal expansion by therapeutic pressure, and molecular analysis of CTCs supports the development of optimal therapeutic regimens. (**C**) Various factors related to extravasation, enrichment, and colonization of distant organs for metastasis of CTCs have been unraveled. (**D**) Understanding the mechanisms of drug resistance by CTC characterization contributes to finding more suitable therapies for disease relapse.

Molecular and genomic characterization of CTCs represents a more promising strategy in several aspects of precision medicine. Until recently, immunohistochemical staining, reverse-transcription polymerase chain reaction (RT-PCR), or digital-droplet PCR (ddPCR) have been widely used to characterize CTCs. More sensitive technologies, including single-cell whole-exome sequencing (WES), or single-cell RNA sequencing (sc-RNA Seq), are now being validated as more decisive tools for CTC profiling. For precision and personalized therapy, patient-derived xenograft (PDx) models are explored in preclinical studies. However, the generation of PDx from tissue biopsies is laborious and risky for patients. CTC collection via liquid biopsies, on the other hand, is minimally invasive, less labor-intensive, and has the potential to reflect the patient’s tumor status in real time. Ex vivo culture of CTCs and genome sequencing have demonstrated new therapeutic targets via drug sensitivity testing in breast cancer [121]. In addition, generating CTC-derived explant (CTX) models showed the utility of drug screening through multiomics analysis and biomarker profiling in small-cell lung cancer (SCLC) [122]. Biomarker information generated from enriched CTCs can support effective drug screening and therapeutic drug target identification (Figure 2A).

CTCs can reflect a malignant tumor’s real-time status and are likely to have a different molecular or genomic signature from the primary tumor, mainly due to selection pressures by anti-cancer drugs, which induce genetically distinct subclonal and clonal expansion of cancer cells (Figure 2B). Molecular analysis of CTCs can reveal the clonal evolution caused by therapeutic pressure [123,124]. Thus, repeated CTC sampling can guide optimal treatment regimens based on the molecular profile of evolving tumor burden within individual patients.

CTCs exhibit either epithelial or mesenchymal phenotypes and sometimes an intermediate phenotype assumed to be a metastatic precursor shed from primary tumors. A switch in the molecular signature of the CTCs between epithelial- and mesenchymal-like character in response to therapy has been reported previously [125]. A reduction in the number of CTCs with a more epithelial-like phenotype was observed when the patient was responding well to the treatment. During the course of therapy, when the disease became a progressive disease, a switch to a more mesenchymal-like CTC was observed, implicating the potential of mesenchymal-like CTCs in predicting disease progression and response to therapy [126]. Furthermore, studies on the biology of CTCs have begun to elucidate the molecular mechanisms of CTC enrichment and metastasis pathways including extravasation, survival, and colonization of distant organs [127,128,129]. Various tumorigenic pathway-related molecules have been shown to be enriched in CTCs (Figure 2C).

Information from CTC characterization can be a cutting-edge tool in deciphering the mutations, genomic aberrations, and alteration in oncogenic pathways contributing to tumor progression and resistance to therapy. In anaplastic lymphoma kinase (ALK) rearranged NSCLC, single CTC sequencing revealed multiple mutations in ALK independent pathways (*EGFR*, *KRAS*, *BRAF*, and *TP53*) and two *ALK* mutations (*ALK*^G1202R/F1174C^ and *ALK*^G1202R/T1151M^) associated with acquired resistance to ALK inhibitors [130]. In BC, multiple novel *HER2* mutations identified by plasma genotyping showed resistance to multiple targeted therapies [131]. To find more suitable therapies for recurrent disease, understanding drug-resistant mechanisms is pivotal (Figure 2D).

With an increasing focus on targeted therapy, it is important to interrogate CTCs for targetable alterations, and a treatment decision based on the molecular signature of the CTCs might prove to be more beneficial to the patients. Unlike conventional cytotoxic therapies, it is increasingly important to identify suitable pharmacodynamic and predictive biomarkers for targeted therapy.

#### 3.2.1. HER2^+^ CTCs as a Molecular Biomarker for Treatment Prediction

HER2 is overexpressed in approximately 20–25% of breast and gastric cancers and is a top-tier biomarker for deciding the treatment strategy [132]. Discordance between the molecular signature of the primary tumor and CTCs is a major concern in targeted therapy, and studies have shown that a relatively large subset of HER2^−^ mBCs show HER2^+^ CTCs [133]. A prospective multicenter trial on mBC patients observed a discordance in HER2 expression between the primary tumor and CTC in 18.8% of patients. The rate of BC patients with HER2^−^ primary tumors and HER2^+^ CTCs was 32% and 49%, as determined by two different assays for CTC detection [134]. A separate study showed that up to 52% of the HER2^+^ patients had HER2^−^ CTCs and did not show significant improvement in the median PFS upon receiving anti-HER2 therapy [135]. A similar study by Zhang et al. showed that patients with HER2^−^ CTCs do not benefit from anti-HER2 therapy despite having a histologically HER2^+^ mBC [136].

The clinical utility of *HER2*-amplified CTCs in HER2^−^ BC was evaluated in the CirCe T-DM1 trial. This prospective phase 2 trial assessed the efficacy of anti-HER2 therapy using trastuzumab-emtansine (T-DM1) in a single-arm study without comparing it with any other therapeutic regimen in HER2^−^ mBC patients with HER2^+^ CTCs. The limited number of CTCs with *HER2* amplification in this study suggested that the *HER2*- amplified CTC is not a major player in modulating tumor burden and is not a CTC subclone that shows exponential proliferation during therapy [137]. The study also concluded that the competency of T-DM1 treatment in patients who are histologically HER2^−^ with *HER2*-amplified CTC could not be completely ruled out. At the same time, the EORTC 90091-10093 BIG 1-12 Treat CTC trial showed that trastuzumab does not decrease the detection rate of CTCs in *HER2*-non-amplified early BC patients [4,138,139,140].

The DETECT-III trial is also investigating the efficacy of HER2-targeted therapy in HER2^−^ patients with HER2^−^ CTCs as well as the significance of CTC as an early predictive biomarker for treatment response. The study had a different approach to the CirCe T-DM1 trial and compared lapatinib as HER2-targeted therapy in combination with chemotherapy versus chemotherapy alone in the patient group [134,139,140]. A proportion of HER2^−^ BC patients persistently have only HER2^−^ CTCs, and the DETECT-IV trial is investigating the therapeutic strategies for this group. The results for these trials are awaited and would help us better understand the clinical utility of HER2^+^ or HER2^−^ CTCs as a predictive biomarker for mBC. HER2-targeted therapy has also been shown to have a significant impact on CTC status.

In mBC, patients with ongoing anti-HER2 therapy have lower overall CTC levels than when treated with chemotherapy and endocrine therapy and should be considered when comparing different therapy groups [141]. These studies emphasize the assessment of HER2 status in CTC irrespective of the HER2 status of the primary tumor, a routine practice, and underscore that such assessment could help identify the patient groups that would benefit from the therapy. Further research with larger cohorts is required to validate the clinical utility of HER2 assessment in CTCs for treatment selection. In addition, it should be noted that the methodology for HER2 assessment in CTCs varies for different studies: the most widely used methods include immunocytochemistry staining of CTCs, and highly sensitive RT-PCR-based techniques [133,134]. Meanwhile, there is no optimal cutoff for HER2 positivity in immunofluorescence or fluorescence in situ hybridization-based assays, resulting in varied results on the prognostic or predictive impact of HER2^+^ CTCs. A consensus on the optimal cutoff combined with the use of multiple biomarkers downstream of HER2 signaling might be necessary to obtain a better insight into the clinical utility of this biomarker.

#### 3.2.2. EGFR Expression on CTCs

EGFR expression is associated with the EMT of cancer cells and the motility, migration, and survival of CTCs, in addition to being an important biomarker in the context of targeted therapy [142,143]. Studies have evaluated EGFR expression in CTCs by immunostaining methods or RT-PCR, whereas *EGFR* mutations are evaluated based on dd-PCR or next-generation sequencing (NGS). *EGFR* mutations or activation are also critical players in acquired resistance to anti-EGFR therapy [144,145]. The first study that evaluated the predictive potential of EGFR expression in CTCs from CRC patients indicated that EGFR expression in CTCs does not predict the response to EGFR-targeted therapy using cetuximab [146]. In a study on mCRPC patients who had experienced treatment failure with androgen deprivation therapy and had received docetaxel, CTCs were demonstrated to be an independent predictor for OS. The EGFR expression on CTCs also proved to be a potential tool for assessing the response to chemotherapy and to predict the disease outcome in mCRPC patients [147]. In non-metastatic BC patients with CK-negative EGFR-positive CTCs, poor PFS post-therapy has been observed; however, the relatively smaller number of patient samples analyzed makes it difficult to derive any clear predictive role of EGFR expression [148]. EGFR expression has also been observed on CTCs from soft tissue sarcoma patients opening new ground for research with promising potential [149]. Studies have also evaluated the expression of phosphorylated EGFR by immunostaining in CTCs from BC patients and demonstrated the activation of these signaling kinases in CTCs [150].

Mutations in *EGFR* are known to impact the outcome of anti-EGFR therapies in LC [151,152]. Exon 19 deletion and L858R can predict the sensitivity to treatment using erlotinib and gefitinib, whereas the T790M mutation of exon 20 is associated with acquired resistance to these drugs [153,154,155]. With the FDA approval of osimertinib, a third-generation EGFR inhibitor which is now the standard front-line therapy for *EGFR*-mutant NSCLC, a noninvasive tool to detect secondary *EGFR* mutations has become imperative. Analyzing the *EGFR* oncogenic driver mutations in CTCs has taken center stage of EGFR research in CTCs, and thanks to highly sensitive mutation analysis methods such as multiplex-qPCR, NGS, and ddPCR, it is hoped to transform the concept of precision medicine in *EGFR*-mutated cancers. Strong concordance between the *EGFR* mutations in CTCs and matched primary tumor samples have been reported previously [115,143]. However, a study by Sundaresan et al. compared T790M mutation from tissue biopsies with simultaneously collected CTC and circulating tumor DNA (ctDNA) samples from NSCLC patients and observed discordance from tissue samples in 35% of patients [156]. It needs to be noted that the discordance observed in the samples could also have arisen due to technological limitations, insufficient sample amounts for LBx, and sampling different tumor cell populations. Serial increase in CTC counts is correlated with disease progression with the emergence of additional *EGFR* mutations in some cases. However, combining alternate methods such as other LBx biomarker assays from ctDNA and exosomes samples may improve the clinical utility of *EGFR* mutation detection in CTCs, and in the future, be a surrogate for tissue EGFR testing [157,158].

#### 3.2.3. AR-V7 Expression on CTCs

Androgen receptor splice variant 7 (*AR-V7*) is a splice variant of androgen receptor (AR) mRNA and is emerging as a biomarker for AR signaling (ARS) inhibitor treatment in mCRPCs. The decision to administer ARS inhibitors or taxanes in mCRPC patients is a critical one, and the need for a biomarker that can predict the response or outcome of these treatments is medically unmet [159,160]. *AR-V7* mRNA was detected by RT-PCR in CTCs isolated from patients with CRPC and showed that the presence of the *AR-V7* was associated with resistance to enzalutamide and abiraterone [161]. Another study from the same group demonstrated that detection of AR-V7 in CTCs is not associated with primary resistance to taxane therapy. In patients with AR-V7-positive (AR-V7^+^) CTCs, taxanes were more beneficial than ARS inhibitors, whereas in AR-V7-negative (AR-V7^−^) patients, taxanes and ARS inhibitors were shown to have comparable efficacy [162]. An increase in the incidence and burden of AR-V7^+^ CTCs has been shown to increase during PC progression and promote androgen depletion-resistant growth, suggesting AR-V7 expression as an adapted response to systemic therapy [163,164,165]. A study by Dr. Howard Scher et al. evaluated the use of AR-V7 expression on pre-therapy CTC in predicting the response to treatment. The study focused on the treatment decision points in the management of individual patients. Patients who show a progressive disease require a change in systemic therapy with approved standard of care drugs. Scher et al. showed that patients harboring pre-therapy AR-V7^+^ CTCs have a better OS with taxanes than with ARS inhibitors [165]. A follow-up study confirmed that the presence of nuclear-localized AR-V7 in CTCs might predict better survival with taxane therapy compared to ARS inhibitors in mCRPC. A diagnostic test that helps physicians make an informed decision on treatment selection between taxanes or ARS inhibitors can potentially improve the outcome. A series of studies recently have published similar results and clinically validated AR-V7^+^ CTCs in mCRPC as a predictor for resistance to ARS-directed therapies and not indicative of resistance to taxanes [166,167,168,169]. An additional treatment option for AR-V7^+^ CTC patients who show resistance to enzalutamide and abiraterone needs to be clinically validated. The PROPHECY study evaluated the detection of *AR-V7* mRNA in CTCs and showed that it is associated with shorter OS and PFS when patients are under an anti-androgen therapy regime [170]. Currently, CABA-V7, a single-arm phase 2 multicenter trial, is investigating the response to cabazitaxel, a next-generation taxane treatment in mCRPC patients with AR-V7^+^ CTCs. The IMPROVE trial, a phase II two-arm trial, is assessing the efficacy of a combination first-line treatment with enzalutamide and metformin in mCRPC patients under androgen deprivation therapy and is also analyzing the potential clinical use of AR-V7 expression on CTCs in treatment outcomes [171]. Making AR-V7 CTC tests a routine clinical assay can enable selecting patients who are most likely to respond to a particular therapeutic regimen while avoiding patient morbidity and cost associated with a treatment that may not be beneficial [172,173].

#### 3.2.4. MET Expression on CTCs

MET is a receptor tyrosine kinase that is known to play a critical role in cell proliferation. *MET* amplification and overexpression are correlated with poor prognosis, metastasis, and resistance to therapy in several cancer types, including NSCLC, CRC, renal cancers, gastroesophageal tumors, and ovarian cancer. *MET* amplification has also been identified as a mechanism of resistance to EGFR-targeted therapies [174,175,176,177,178]. MET-targeted therapies are considered an important treatment strategy in *MET*- amplified and MET-overexpressed cancers. In head and neck squamous cell carcinoma (HNSCC) patients receiving nivolumab treatment, MET-positive CTCs have been shown to be correlated with shorter OS [179,180]. Several MET-targeting monoclonal ABs and small molecule inhibitors have entered clinical evaluation, and highly selective MET inhibitors such as capmatinib and tepotinib have gained FDA approval. Agents targeting MET proved to be effective in patients with *MET* exon14 deletion and are expected to be effective in *MET* amplification or MET overexpression; however, there is no consensus on the methods to determine *MET* amplification in the clinical setting. MET expression and alterations have been detected in CTCs and ctDNA from cancer patients by immunostaining, RT-PCR, dd-PCR or sequencing technologies [17,181,182], and might prove to be a promising strategy in identifying a biomarker to select a MET-addicted tumor that would benefit significantly from anti-MET targeted therapy.

Zhang et al. employed DNA-FISH for *MET* amplification analysis and demonstrated the method to be 80% sensitive for *MET*-amplified cancers, 40–80% sensitive for MET-overexpressed cells and 100% sensitive for MET-negative CTCs [182]. NGS or ddPCR approaches to detect *MET* alterations are also being widely investigated in ctDNA and CTC samples from MET-addicted tumors [183]. Recently Novartis’s MET inhibitor capmatinib (Tabrecta^®^) became the only drug to have FDA approval for metastatic NSCLC patients with *MET* exon 14 skipping mutation [184], and the liquid biopsy-based testing for mutation by FoundationOne^®^LiquidCDx also gained approval for use as a CDx for patient selection for Tabrecta^®^ therapy. A recent study investigated a novel small molecule MET inhibitor, ABN401, for its efficacy as a selective therapeutic strategy based on diagnostic biomarker tests in MET-addicted cancers [185]. This inhibitor is now in a phase I-II dose-escalation study and is being planned to be used to develop a CDx product using *MET* amplification and exon 14 skipping from CTCs and cfDNA as a biomarker for patient selection and treatment response evaluation.

#### 3.2.5. PD-L1 Expression on CTCs

Programmed death-ligand 1 (PD-L1) expression is associated with the treatment efficacy of immune checkpoint inhibitors (ICIs) in different cancer types. The benefit from ICIs in the clinic largely depends on appropriate patient selection based on predictive biomarkers, making the need for a reliable biomarker urgent. CTCs are a noninvasive, surrogate sample accessible to longitudinal sampling and could provide a snapshot of PD-L1 status in patients. Detection of PD-L1 positive (PD-L1^+^) CTCs and their prognostic potential have been demonstrated in NSCLC [186,187,188,189,190,191,192,193], BC [194], urothelial carcinoma [195], gastric cancer [196], cholangiocarcinoma [17], and melanoma [197]. Dynamic changes in the expression of PD-L1 in response to radiotherapy have also been reported in a study in NSCLC [191]. The proportion of PD-L1^+^ CTCs increased significantly in response to radiation and was associated with a poor prognosis. An interesting observation in this study was that one of the patients with high PD-L1^+^ CTCs at visits two and three was administered with pembrolizumab after the initial progression and remained stable for seven months, indicating that the patients who are likely to become sensitized to ICI treatment could be identified using PD-L1 expression on CTCs [191]. A study by Kallergi et al. showed the predictive potential of programmed cell death protein-1 positive (PD-1^+^) CTCs in NSCLC patients receiving chemotherapy. Though both PD-1^+^ and PD-L1^+^ CTCs could be identified at baseline and after chemotherapy, the study showed that patients with > three PD-1^+^ CTCs at baseline had shorter median PFS, suggesting a potential role for PD-1^+^ CTCs [190]. Ilie et al. showed poor clinical outcomes in patients with PD-L1^+^ CTCs receiving first-line chemotherapy [198]. Notably, studies have observed a decrease or no change in CTCs in patients who show a good response to treatment using ICIs—pembrolizumab, nivolumab, or atezolizumab—whereas all patients showed an increase in the number of PD-L1^+^ CTCs upon disease progression [186]. Co-expression of PD-L1 with mesenchymal-like CTCs was shown to be associated with poor survival of NSCLC patients, indicating that malignant disease progression is driven by EMT and PD-L1 expression, emphasizing the relevance of PD-L1 profiling for patient selection for immunotherapies or combination therapies [187]. In gastric cancer patients, PD-L1 expression on cell surface VIM-positive CTCs was associated with shorter OS and poor therapeutic response. A recent study showed that PD-L1^+^ CTCs are an independent predictive biomarker for clinical benefit and therapeutic response to pembrolizumab in advanced melanoma patients. This pilot study showed that patients with PD-L1^+^ CTCs had significantly longer PFS, treated with pembrolizumab, compared to patients who had PD-L1 negative (PD-L1^−^) CTCs [197].

Studies on the predictive significance of PD-L1 are emerging, with several ongoing clinical trials investigating the potential use of PD-L1^+^ CTCs in patient selection and evaluating the outcome of treatment with ICIs. In addition, several of these trials are investigating the feasibility of PD-L1 expression analysis on CTCs using various isolation technologies. For example, the IMMUNO-PREDICT trial is investigating the feasibility of using the ISET^®^ technology. The I-CURE-1 (NCT03213041) trial is evaluating the impact of combined treatment with carboplatin and pembrolizumab in patients with CTC-positive HER2^−^ mBC previously treated with anthracyclines and taxanes. As a tertiary objective, the study is investigating the immune biomarker PD-L1 in CTCs isolated using the CellSearch^®^ method, and its correlation with therapeutic benefit (NCT03213041). Results from these studies would provide a better understanding of the clinical use of PD-L1 in CTCs as a predictive biomarker for ICI treatment in different cancer types. Table 2 summarizes the clinical trials evaluating the predictive potential of CTCs.

### 3.3. Genomic Analysis of CTCs for Their Role as a Predictive Biomarker

Genomic analysis of CTCs allows the analysis of mutations and genetic alterations contributing to intratumor heterogeneity, clonal evolution, and drug resistance [199]. Analysis of genomic alterations that can help guide the selection of targeted therapy is the most promising clinical application of genomic analysis of CTCs. However, only a few studies have looked at mutations of CTCs, as ctDNA analysis is a more preferred strategy for mutation analysis from LBx samples [200]. Nevertheless, ctDNA-based analysis is limited to point mutations, gene rearrangements, copy number alterations, and DNA methylations, whereas CTC-based analysis allows DNA, RNA, and protein-based molecular and genomic profiling. For mutation analysis of CTCs, an amplification step should be employed to ensure enough quantity of genome for analysis. Most widely used techniques include whole-genome amplification (WGA) and degenerate oligonucleotide-primed PCR (DOP-PCR) for genome amplification and whole transcriptome amplification techniques such as SMART-seq [201], Quartz-seq [202], and STRT-seq [203,204]. The genomic material obtained is subjected to sequencing or mutational analysis using NGS or PCR techniques [204,205].

Wang et al. compared the genetic mutations in CTCs and their matched tumors, as well the change in mutational status in CTCs before and after treatment. For mutations detected in a single gene, the concordance was 53.05%. Heterogeneity in mutational status existed at the single cell level of CTCs, and the mutational status of CTCs was discordant with that of matched tissue biopsy samples [206]. The mutation signature of CTCs provided reliable information on the tissue of origin and mechanism of tumor development and could suggest potential treatment options in a study by Gulbahce et al. [207]. Whole-genome sequencing based on fragment read technology was utilized for mutation analysis, and driver mutations and tissue of origin of the cells were identified in the whole genomes of 34 CTCs from a patient with mBC. Acquiring novel mutations in CTCs in response to therapy was reported in BC patients receiving chemotherapy. The study also identified a mutation in the *ERBB2* gene (p.V777L), which could have played a critical role in resistance to therapy as this mutation was identified in all post-therapy CTCs [208]. Ni et al. demonstrated in their study that copy number variations (CNVs) are specific to cancer types, reproducible from cell to cell and even from patient to patient. Clinical relevance of *PIK3CA*, *RB1*, and *TP53* mutations in erlotinib drug resistance was demonstrated, implicating the relevance of CTC sequencing in predicting treatment response. The study also demonstrated that single nucleotide variations (SNVs) across CTCs vary with time in response to first-line and second-line chemotherapy. Interestingly, CTC CNVs were not affected by drug treatments, suggesting the potential of CTC genomic analysis in noninvasive diagnostics [209]. Scher et al. showed that analysis of all CTC sub-populations might hold clinical relevance. mCRPC driver mutations were predominantly present in CTC sub-populations of patients showing resistance to therapy. Non-traditional and rare CTC subtypes including CK-negative and small CTCs were demonstrated to have mutations responsible for therapeutic resistance [210]. D’Oronzo et al. employed DEPArray^TM^ technology to sort epithelial-like and mesenchymal-like CTCs and compared genomic heterogeneity with matched tissue biopsies. The study showed that mesenchymal-like CTCs were the most heterogeneous CTC subtype and postulated that the mutations detected in all CTC subsets might be considered as genomic biomarkers of metastatic dissemination [211]. Copy number analysis of CTCs from advanced PC, NSCLC, and BC patients has demonstrated that single-cell genomic analysis provides a window for tumor heterogeneity analysis and tumor evolution in response to therapy [123,212,213]. In advanced NSCLC WES of CTCs, and comparison of mutation profiles in primary and progressive tumor specimens reflected different evolutionary mechanisms in CTCs and lymph node metastasis [214]. Mutational anlaysis of CTCs using these novel technologies can be explored for identification of drug resistance mechanisms and novel druggable targets in cancer patients receiving therapies.

Studies on the predictive potential of genomic CTC analysis are still in their infancy and need to undergo extensive clinical validation. A major setback in the genomic analysis of CTCs is represented by the technical challenges associated with successful genome amplification and library preparation [215]. All approaches for CTC WGA have their pros and cons. The DOP-PCR methodology has a low genome coverage, making it unsuitable for SNV analysis, whereas the multiple-displacement amplification (MDA)-based WGA method is often associated with allelic dropouts (ADO), making CNV studies difficult. The high false-positive rate of multiple annealing and looping-based amplification cycles (MALBAC) makes it unsuitable for point mutation analysis [216,217]. In a recent study, Lu et al. evaluated four WGA/NGS workflows for genomic analysis of CTCs; PCR-based (GenomePLex and Ampli1), MDA (Repli-g), hybrid PCR, and MALBAC. Their comprehensive analysis showed that MALBAC WGA coupled with low-pass whole-genome sequencing is an optimal workflow for genome-wide CNV profiling at a single-cell level. This study demonstrated the drawback of the current single-cell CTC analysis workflows in mutation analysis and concluded that none of the WGA methods could achieve sufficient sensitivity and specificity for genome-wide mutation analysis at a single-cell level [218]. Table 3 summarizes the FDA-approved LBx tests for CDx.

CTC morphology and chromosome instability in CTCs is expected to carry prognostic/predictive information. In a study in NSCLC patients, small CTCs with irregular nuclei showed a significantly increased risk of disease relapse, implicating the feasibility of morphological classification of CTCs in patient stratification [219]. The recently revealed data from the *CARD trial* revealed an interesting aspect of morphologic analysis of CTCs. This study on mPC patients shows that patients with CTCs harboring chromosomal instability also had increased morphological diversity of CTCs, and were associated with shorter OS and radiological PFS (rPFS) in those treated with cabazitaxel [220].

**Table 2:** Clinical trials evaluating the predictive role of circulating tumor cells.

Trial	Disease Condition	Purpose	Phase	Trial Result	Ref.
REMAGUS-02	Localized HER2^+^ mBC	Determine if CTCs were present in patients receiving neoadjuvant chemotherapy before initiation of chemotherapy and at the end of chemotherapy before surgery	III	CTCs can be detected in the blood of patients with large operable or locally advanced breast cancers before initiation of neo-adjuvant chemotherapy and can be monitored during treatment	[79]
DETECT-III NCT01619111	HER2^−^ mBC	Evaluate the efficacy of HER2-targeted therapy in patients with mBC and HER2 positive CTCs	III	HER2 positive CTCs can be detected in a relevant number of patients with HER2 negative tumors and will be mandatory to correlate the assay-dependent HER2 status of CTCs to clinical response on HER2 targeted therapies	[134]
SWOG S0500 NCT00382018	mBC	Study treatment decision-making based on blood levels of tumor cells in women with mBC receiving chemotherapy	III	Confirmed the prognostic significance of CTCs in patients with mBC receiving first-line chemotherapy. Early switching to an alternative cytotoxic therapy was not effective in prolonging OS	[90]
TREAT CTC NCT01548677	HER2^−^ mBC	Efficacy study of Herceptin to treat HER2-negative CTC breast cancer	II	Real-time screening of patients with early breast cancer for CTCs is feasible (pilot phase results)	[138]
STIC-CTC NCT01710605	HR^+^ HER2^−^ mBC	Analyze the CTC count (CellSearch^®^)-driven first-line treatment choice	III	CTC count may be a reliable biomarker method for guiding the treatment choice between chemotherapy and endocrine therapy as the first-line treatment choice	[114]
Circe T-DM1 NCT01975142	HER2^−^ mBC	Evaluate the validity of HER2-amplified CTCs to select mBC considered HER2^−^ for trastuzumab-emtansine treatment	II	CTCs with HER2 amplification can be detected in a limited subset of HER2^−^ mBC and treatment with T-DM1 achieved partial response	[137]
CABAV7 NCT03050866	mCRPC	Efficacy of cabazitaxel in mCRPC patients with AR-V7^+^ CTC	II	Awaited (prospective validation is needed to investigate if AR-V7 could fulfil the criteria as a predictive biomarker)	[169]
PROPHECY NCT02269982	mCRPC	Evaluate circulating tumor-derived products (CTCs) as a prospective predictor in higher risk mCRPC in the context of AR-directed therapies	NA	Detection of AR-V7 in CTCs is associated with shorter PFS and OS with abiraterone or enzalutamide, and such mCRPC patients should be offered alternative treatments	[170]
PERSEVERE NCT04849364	TNBC	Evaluated the association of ctDNA and CTCs after neoadjuvant chemotherapy with disease recurrence	II	Detection of ctDNA and CTCs in patients with early stage TNBC after neoadjuvant chemotherapy was independently associated with disease recurrence	[81]
CARD NCT02485691	mCRPC	Evaluated the impact of CTC morphologic subtypes prior to treatment in CARD trial	IV	Presence of chromosome instability at screening is associated with increased CTC morphological diversity, and also had poor rPFS and OS, when treated with cabazitaxel	[220]
CTCNeoBC NCT03732339	Locally advanced BC	Evaluate the predictive value of CTC using GILUPI cell collector	NA	Awaited	NA
PROLIPSY NCT04556916	PC	Assess the validity of CTC and tumor cell products for early prostate cancer detection	NA	Awaited (Not yet recruiting)	NA
I-CURE NCT03213041	HER2^−^ mBC	Evaluate the impact on PFS with carboplatin-pembrolizumab combination in patients with CTC-positive HER2-negative mBC previously treated with anthracyclines and taxanes	II	Awaited (Recruiting)	NA
IMMUNO-PREDICT NCT02827344	LC	Demonstrate the feasibility of the analysis of PD-L1 expression on CTCs	NA	Awaited (Recruiting)	NA

This table shows the major clinical trials that are/have investigated the predictive role of CTCs in different cancers. mBC: metastatic breast cancer, BC: breast cancer, mCRPC: metastatic castration-resistant prostate cancer, PC: prostate cancer, LC: lung cancer.

**Table 3:** FDA-approved liquid biopsy tests for companion diagnostics.

Companion Diagnostic Test	Drugs	Disease Type/ Sample Type	Mutation	Technique for Mutation Detection	Manufacturer
cobas EGFR mutation test	erlotinib (Tarceva), osimertinib (Tagrisso), gefitinib (Iressa)	NSCLC/plasma	*EGFR* mutations (exon 19 deletions, L858R mutation), T790M mutation	Real time polymerase chain reaction (RT-PCR)	Roche
Guardant 360 CDx	osimertinib (Tagrisso)	NSCLC/lasma	*EGFR* mutation	Next-generation sequencing	Guardant
	amivantamab (Rybrevant)		*EGFR* mutation (exon 20 mutation)		
FoundationOne Liquid CDx	rucaparib (Rubarca)	Ovarian cancer/plasma	*BRCA1*, *BRCA2*	Next-generation sequencing	Foundation One
	alectinib (Alecensa)	NSCLC	*ALK* rearrangement		
	alpelsib (Piqray)	Breast cancer	*PIK3CA*		
	olaparib (Lynparza)	mCRPC/plasma	*BRCA1*, *BRCA2*, *ATM*		
	rucaparib (Rubarca), AR-directed therapy, Taxane	mCRPC/plasma	*BRCA1*, *BRCA2*		
	gefitinib (Iressa), osimertinib (Tagrisso), erlotinib (Tarceva)	Lung cancer	*EGFR* mutations (exon 19 deletions, L858R mutation)		
	capmatinib (Tabrecta, Novartis)	NSCLC	*MET* (exon 14 mutations)		
therascreen PIK3CA RGQ PCR Kit	alpelsib (Piqray)	Breast cancer/plasma	*PIK3CA*	Real-time polymerase chain reaction (RT-PCR)	Qiagen GmbH
ArcherDx’s ArcherMET Assay	tepotinib (Tepmetko, Merck KGaA)	NSCLC/plasma	*MET* (exon 14 skipping)	Next-generation sequencing	Archer

## 4. Limitations in CTC-Based Studies

The concept of precision medicine aims at providing individual cancer patients with appropriate cancer management, starting from disease screening and selecting the right drug through to monitoring disease relapse or recurrence, and requires the integration of single-cell isolation techniques, genomics, proteomics, pharmacogenomics, and pharmacodynamics studies [221]. With the advent of novel technologies for CTC isolation and analysis, the clinical utility of CTCs is being investigated and appreciated more than ever before. Rapid progress in basic cancer research and results from the studies investigating the prognostic and predictive potential of CTCs in a clinical setting are expected to provide an effective way to implement cancer precision medicine [222]. The breakthrough study by Hamza et al. developed a technique to measure the kinetics and potency of CTCs for the very first time. The researchers were able to measure CTCs in real time by exchanging CTC-containing blood between tumor-bearing mice and healthy mice. This approach gave insight into the intravasation rate and half-life times of CTCs in circulation, and is hoped to widen our understandings of the role of CTCs as a rate-limiting step in metastasis [223].

A major limitation in translating CTC studies to clinical practice lies in the lack of approved robust technologies for CTC isolation. Even though more than a decade has passed since the FDA approval for the CellSearch^®^ system, it is the only approved CTC enrichment system. The rarity of CTCs in circulation impedes their efficient isolation; however, the advent of novel technologies has enabled CTC isolation with high sensitivity and specificity at a higher yield, and more systems with FDA approval are anticipated in the coming years. One such promising approach is the intravenous collection of CTCs using indwelling catheter-based systems [41,43,44,224]. These technologies allow the interrogation of larger blood volumes compared to the routine phlebotomy specimens and require further validation and approval to facilitate their clinical use.

Further, a major concern in most clinical trials for which some results have been published is the significantly small number of CTCs that are detected, in addition to the lack of consensus over the cutoff for the expression level of CTC-specific biomarkers. For example, in studies that evaluated HER2 expression on CTCs, a single HER2^+^ CTC has been shown to have clinical significance in certain studies, whereas a cutoff of ≥ 10 CTCs has also been reported [225]. In immunocytochemistry-based HER2 expression analysis, the scoring of cells as HER2^+^ is also very ambiguous between different study groups, making it difficult to compare the results from different studies [134,226,227]. All these highlight the need for a method that has universal consensus through an appropriately designed prospective study.

Another hindrance to CTC analysis in clinical practice is the heterogenic nature of CTCs [228]. CTC frequency and molecular signature show huge variation within the same cancer type and between cancer types [229], resulting in the misjudgment of CTC enumeration in the study samples. A vast majority of clinical studies use EpCAM-based CTC enrichment systems, as they have been demonstrated to have clinical validity in the prognosis of several solid tumors. The CTC detection rate in the most widely used CellSearch^®^ system is from 20–50% in advanced cancers, mostly owing to the dynamic heterogeneity of CTCs. This limitation in CTC detection rate, combined with inter-patient variability, has had a huge impact on clinical trial design and the success rate of these trials, resulting in the need to include large patient cohorts for each study. Moreover, EMT is a major molecular phenomenon associated with metastasis, and this has raised questions on using EpCAM as a universal biomarker for CTC enrichment [230,231,232,233]. It is likely that other CTC sub-populations (non-EpCAM) encompass the metastatic founder cells and may have a critical role in clinical disease progression [125,126,234]. A remarkable association between the expression of mesenchymal-like markers and CTC clusters, disease prognosis, and response to therapy has been demonstrated [235,236,237,238]. EMT-based CTC enrichment and characterization of therapeutically relevant biomarkers is expected to facilitate tailored cancer management and guide treatment decisions.

Furthermore, systems that use the biological properties of CTCs for their enrichment assume that CTCs express or do not express certain biomarkers. A general drawback here is that the CTC subsets that do not express these biomarkers are not enriched [64,65]. Label-free CTC isolation methods or employing multiple biomarkers for CTC enrichment are the strategies employed to overcome this issue [239]. Most of these strategies are in translational clinical studies and need to be validated in large clinical cohorts. Further improvement of detection technologies combining various inherent properties of CTCs would optimize CTC selection. In addition, using the right technique that is optimal for the study design, cancer type, and its clinical application would also have an impact.

Single-cell level CTC analysis is now the focus of several CTC studies, and molecular and genomic characterization of CTCs at the single-cell level has revealed genomic variations or sub-clonal mutations specific to CTCs [240,241,242]. SNVs during the course of chemotherapy, CNV profiles that can determine chemo-sensitive and chemo-refractory diseases, and the molecular signature for monitoring response to therapy have been identified in single-cell CTC studies [209,212,213,243,244,245]. CTC isolation at the single-cell level is, however, a tedious process and, in most cases, requires a pre-enrichment step and subsequent single-cell sorting. Techniques such as laser capture microdissection [246] and single-cell sorting using DEPArray^TM^ [75,76,247], CellCelector^TM^ [248], VyCAP’s or Puncher technology [249] have demonstrated usefulness for single-cell CTC analysis. Downstream analysis of CTCs at the genomic level relies on single-cell profiling technologies such as whole-genome/exome sequencing, single-cell RNA sequencing, secretome profiling, etc. Most techniques require a WGA/whole transcriptome amplification step prior to sequencing. However, to achieve high sensitivity and specificity for the analysis of mutations and CNV, specific performance metrics and analytical methods must be employed. Applying these techniques in a clinical perspective is limited as CTCs obtained at a single point alone may not represent dynamically changing CTCs. Furthermore, the quality and the number of CTCs isolated and the sample size in some of these studies are not sufficient that the molecular findings from these studies may only be relevant for the small sub-population in which the study was conducted. A universal workflow for single-cell analysis of CTCs is imperative for their proper implementation in clinics and is also expected to answer questions on tumor heterogeneity and clonal evolution exerted by chemotherapy agents.

## 5. Conclusions and Future Perspectives

The relevance and promise of CTC screening and analysis in predicting cancer progression and response to therapy are implicated in several studies. Novel techniques and clinical utilities are added to the repertoire every day as a result of extensive research in this area. Though this paper reviewed only CTCs as an LBx, it is evident from several studies that combining information from several LBx markers, including ctDNA, exosomes, circulating micro RNAs, etc., can provide a wide spectrum of understanding on their clinical applications. Recently studies have developed protocols and workflow for analyzing multiple biomarkers from a single tube of blood. The data from CTCs and ctDNA are indicative of different cancer cell types in circulation and can provide information that is mutually exclusive and complementary, thereby having the potential to probably provide in-depth information on patients’ disease progression and response to treatment [158]. With the FDA approval for several ctDNA-based CDx tools for cancer diagnosis and response monitoring in a wide range of solid tumors, the focus on developing CTC-based CDx tools has increased exponentially (Table 3). Major limitations in single-cell CTC analysis, including technical, economical, and logistic challenges, need to be addressed and overcome to improve the efficacy of the clinical application of single-cell CTC analysis. Efforts to isolate viable CTCs and culture them for their genetic and epigenetic characterization are ongoing and are expected to be developed into potent tools for personalized drug screening and tailored therapy. Recently it was shown that CTC cluster phenotypes in an ex vivo CTC culture system can provide crucial information on response to treatment [121,122,250,251]. Noninvasive early detection of cancer, as well early identification of therapeutic response, has been achieved in separate studies using LBx [252,253]. By further improving the abovementioned technologies, it might be possible to derive predictive data, which is more clinically relevant and could help rapid translation of CTCs from the benchtop to bedside. Furthermore, the most critical step in making the analysis of CTCs a routine clinical practice is their extensive clinical validation. Proper design of clinical trials and choosing appropriate treatment interventions for the right patient population is essential for the success of the clinical trials, and results from such trials are expected to provide some insights and answer the questions raised regarding the clinical usability of CTCs.

## Figures and Tables

**Table 1:** Selected circulating tumor cell enrichment technologies and devices.

Technique	CTC Isolation Platform	Marker	Capture Efficiency	Recovery	Purity	Viability	Clinical Detection Rate (No. of Patients)	Ref.
Immuno magnetic	CellSearch	EpCAM	-	85%	-	Non-viable	71%	[12,13]
	Magsweeper	EpCAM	62–70%	-	50%	Viable	100%	[14]
	EasySep	CD45 (Negative Selection)	79%	-	42%	-	-	[15]
Immuno magnetic and microfluidic	Isoflux	EpCAM	73–81%	-	-	-	96%	[16]
	GenoCTC	EpCAM, MET, Vimentin	-	77%	90%	Viable	94% (*n* = 16)	[17]
	CTC-iCHIP	EpCAM, CD45+ Size-based sorting	77–98%	-	99%	-	90% (*n* = 41)	[18]
	Magnetic Sifter	EpCAM	74%	-	-	Viable	100% (*n* = 4)	[19]
	OncoBean Chip	EpCAM	90%	-	-	Viable	100% (*n* = 4)	[20]
	LiquidBiopsy	Trop2, Muc1, Her3, MelCAM, EpCAM	-	70–80%	70–77%	-	-	[21]
Microfluidic immunocapture	CEE	Trop1, Trop2, MET, FBP, N-Cadherin, CD318, HER2, MUC1, EGFR, MSCA-1	76%	89%	-	-	63% (*n* = 24)	[22]
	CTC-CHIP	EpCAM	65%	>65%	52–67%	Viable	99%	[23]
	Biofluidica HT_CTC chip	EpCAM, Seprase	95%	90%	>86%	Viable	100% (*n* = 7)	[24,25]
	CytoQuest	EpCAM, Cell Surface Vimentin	-	-	-	Non-viable	-	[26,27]
	Herringbone Chip	EpCAM	80%	-	-	Viable	93% (*n* = 15)	[28,29]
Micro filtration	Parsortix	Size based sorting	45–70%	-	54–69%	Viable	39% (*n* = 26)	[30]
	ISET	Size and deformability-based filtration	-	-	-	Non-viable	80% (*n* = 40)	[31,32,33]
	ScreenCell	Size and deformability-based filtration	-	-	-	Viable	77% (*n* = 76)	[34,35]
Inertial focusing and microfluidics	ClearCell	Size-based	-	-	-	Viable	100% (*n* = 56; *n* = 10)	[36,37]
	VTX-1	Size-based	~80%	54–72%	57–94%	Viable	73–80% (*n* = 15–41)	[38]
Density	OncoQuick	Density fractionation	74–91%	87%	-	Viable	23% (*n* = 61)	[39]
	Accucyte	Density fractionation	-	90%	-	Viable	81% (*n* = 27)	[40]
In vivo immune capture	GILUPI CellCollector	EpCAM	-	-	-	Non-viable	58% (*n* = 108; *n* = 185)	[41,42]
	BPNS- Catheter	EpCAM antibody functionalized catheter	2.1% (in 5 min)	~80%	-	NA	NA	[43]
	Intravascular aphaeretic system	Blood allowed to pass through herringbone graphene oxide chip-coated with anti-EpCAM antibody	80–90%	-	-	Viable	NA	[44]
	MagWire	EpCAM-coated magnetic particle	39–70%	-	-	Viable	NA	[45]

## Data Availability

Not Applicable.

## References

[1] Brown M.V., McDunn J.E., Gunst P.R., Smith E.M., Milburn M.V., Troyer D.A., Lawton K.A. (2012). Cancer detection and biopsy classification using concurrent histopathological and metabolomic analysis of core biopsies. Genome Med..

[2] Ilie M., Hofman P. (2016). Pros: Can tissue biopsy be replaced by liquid biopsy?. Transl. Lung Cancer Res..

[3] Ashworth T.R. (1869). A case of cancer in which cells similar to those in the tumors were seen in the blood after death. Med. J. Aust..

[4] Ignatiadis M., Rack B., Rothe F., Riethdorf S., Decraene C., Bonnefoi H., Dittrich C., Messina C., Beauvois M., Trapp E. (2016). Liquid biopsy-based clinical research in early breast cancer: The EORTC 90091-10093 Treat CTC trial. Eur. J. Cancer.

[5] Alix-Panabieres C., Pantel K. (2016). Clinical Applications of Circulating Tumor Cells and Circulating Tumor DNA as Liquid Biopsy. Cancer Discov..

[6] Dive C., Brady G. (2017). SnapShot: Circulating Tumor Cells. Cell.

[7] Amin M.B., Greene F.L., Edge S.B., Compton C.C., Gershenwald J.E., Brookland R.K., Meyer L., Gress D.M., Byrd D.R., Winchester D.P. (2017). The Eighth Edition AJCC Cancer Staging Manual: Continuing to build a bridge from a population-based to a more “personalized” approach to cancer staging. CA Cancer J. Clin..

[8] Lakhani S.R., Ellis I.O., Schnitt S.J., Tan P.H., van de Vijver M.J. (2012). WHO Classification of Tumors of Breast.

[9] Banko P., Lee S.Y., Nagygyorgy V., Zrinyi M., Chae C.H., Cho D.H., Telekes A. (2019). Technologies for circulating tumor cell separation from whole blood. J. Hematol. Oncol..

[10] Cortes-Hernandez L.E., Eslami S.Z., Pantel K., Alix-Panabieres C. (2020). Molecular and Functional Characterization of Circulating Tumor Cells: From Discovery to Clinical Application. Clin. Chem..

[11] Cho P.Y., Hsieh C.H., Wu M.H. (2020). The combination of Immunomagnetic Bead-based Cell isolation and Optically induced Dielectrophoresis (ODEP)-based microfluidic device for the negative-selection based isolation of circulating tumor cells. Front. Bioeng. Biotechnol..

[12] Allard W.J., Matera J., Miller M.C., Repollet M., Connelly M.C., Rao C., Tibbe A.G., Uhr J.W., Terstappen L.W. (2004). Tumor cells circulate in the peripheral blood of all major carcinomas but not in healthy subjects or patients with nonmalignant diseases. Clin. Cancer Res..

[13] Riethdorf S., Fritsche H., Muller V., Rau T., Schindlbeck C., Rack B., Janni W., Coith C., Beck K., Janicke F. (2007). Detection of circulating tumor cells in peripheral blood of patients with metastatic breast cancer: A validation study of the CellSearch system. Clin. Cancer Res..

[14] Deng G., Krishnakumar S., Powell A.A., Zhang H., Mindrinos M.N., Telli M.L., Davis R.W., Jeffrey S.S. (2014). Single cell mutational analysis of PIK3CA in circulating tumor cells and metastases in breast cancer reveals heterogeneity, discordance, and mutation persistence in cultured disseminated tumor cells from bone marrow. BMC Cancer.

[15] Liu Z., Fusi A., Klopocki E., Schmittel A., Tinhofer I., Nonnenmacher A., Keilholz U. (2011). Negative enrichment by immunomagnetic nanobeads for unbiased characterization of circulating tumor cells from peripheral blood of cancer patients. J. Transl. Med..

[16] Harb W., Fan A., Tran T., Danila D.C., Keys D., Schwartz M., Ionescu-Zanetti C. (2013). Mutational Analysis of Circulating Tumor Cells Using a Novel Microfluidic Collection Device and qPCR Assay. Transl. Oncol..

[17] Chelakkot C., Ryu J., Kim M.Y., Kim J.S., Kim D., Hwang J., Park S.H., Ko S.B., Park J.W., Jung M.Y. (2020). An Immune-Magnetophoretic Device for the Selective and Precise Enrichment of Circulating Tumor Cells from Whole Blood. Micromachines.

[18] Fachin F., Spuhler P., Martel-Foley J.M., Edd J.F., Barber T.A., Walsh J., Karabacak M., Pai V., Yu M., Smith K. (2017). Monolithic Chip for High-throughput Blood Cell Depletion to Sort Rare Circulating Tumor Cells. Sci. Rep..

[19] Earhart C.M., Hughes C.E., Gaster R.S., Ooi C.C., Wilson R.J., Zhou L.Y., Humke E.W., Xu L., Wong D.J., Willingham S.B. (2014). Isolation and mutational analysis of circulating tumor cells from lung cancer patients with magnetic sifters and biochips. Lab Chip.

[20] Murlidhar V., Zeinali M., Grabauskiene S., Ghannad-Rezaie M., Wicha M.S., Simeone D.M., Ramnath N., Reddy R.M., Nagrath S. (2014). A radial flow microfluidic device for ultra-high-throughput affinity-based isolation of circulating tumor cells. Small.

[21] Winer-Jones J.P., Vahidi B., Arquilevich N., Fang C., Ferguson S., Harkins D., Hill C., Klem E., Pagano P.C., Peasley C. (2014). Circulating tumor cells: Clinically relevant molecular access based on a novel CTC flow cell. PLoS ONE.

[22] Mikolajczyk S.D., Millar L.S., Tsinberg P., Coutts S.M., Zomorrodi M., Pham T., Bischoff F.Z., Pircher T.J. (2011). Detection of EpCAM-Negative and Cytokeratin-Negative Circulating Tumor Cells in Peripheral Blood. J. Oncol..

[23] Nagrath S., Sequist L.V., Maheswaran S., Bell D.W., Irimia D., Ulkus L., Smith M.R., Kwak E.L., Digumarthy S., Muzikansky A. (2007). Isolation of rare circulating tumour cells in cancer patients by microchip technology. Nature.

[24] Kamande J.W., Hupert M.L., Witek M.A., Wang H., Torphy R.J., Dharmasiri U., Njoroge S.K., Jackson J.M., Aufforth R.D., Snavely A. (2013). Modular microsystem for the isolation, enumeration, and phenotyping of circulating tumor cells in patients with pancreatic cancer. Anal. Chem..

[25] Dharmasiri U., Njoroge S.K., Witek A.M., Adebiyi M.G., Kamande J.W., Hupert M.L., Barany F., Soper S.A. (2011). High-throughput selection, enumeration, electrokinetic manipulation, and molecular profiling of low-abundance circulating tumor cells using a microfluidic system. Anal. Chem..

[26] Satelli A., Brownlee Z., Mitra A., Meng Q.H., Li S. (2015). Circulating tumor cell enumeration with a combination of epithelial cell adhesion molecule- and cell-surface vimentin-based methods for monitoring breast cancer therapeutic response. Clin. Chem..

[27] Satelli A., Mitra A., Brownlee Z., Xia X., Bellister S., Overman M.J., Kopetz S., Ellis L.M., Meng Q.H., Li S. (2015). Epithelial-mesenchymal transitioned circulating tumor cells capture for detecting tumor progression. Clin. Cancer Res..

[28] Stott S.L., Hsu C.H., Tsukrov D.I., Yu M., Miyamoto D.T., Waltman B.A., Rothenberg S.M., Shah A.M., Smas M.E., Korir G.K. (2010). Isolation of circulating tumor cells using a microvortex-generating herringbone-chip. Proc. Natl. Acad. Sci. USA.

[29] Stott S.L., Lee R.J., Nagrath S., Yu M., Miyamoto D.T., Ulkus L., Inserra E.J., Ulman M., Springer S., Nakamura Z. (2010). Isolation and characterization of circulating tumor cells from patients with localized and metastatic prostate cancer. Sci. Transl. Med..

[30] Hvichia G.E., Parveen Z., Wagner C., Janning M., Quidde J., Stein A., Muller V., Loges S., Neves R.P., Stoecklein N.H. (2016). A novel microfluidic platform for size and deformability based separation and the subsequent molecular characterization of viable circulating tumor cells. Int. J. Cancer.

[31] Hofman V., Ilie M.I., Long E., Selva E., Bonnetaud C., Molina T., Venissac N., Mouroux J., Vielh P., Hofman P. (2011). Detection of circulating tumor cells as a prognostic factor in patients undergoing radical surgery for non-small-cell lung carcinoma: Comparison of the efficacy of the CellSearch Assay and the isolation by size of epithelial tumor cell method. Int. J. Cancer.

[32] Hofman V., Long E., Ilie M., Bonnetaud C., Vignaud J.M., Flejou J.F., Lantuejoul S., Piaton E., Mourad N., Butori C. (2012). Morphological analysis of circulating tumour cells in patients undergoing surgery for non-small cell lung carcinoma using the isolation by size of epithelial tumour cell (ISET) method. Cytopathology.

[33] Farace F., Massard C., Vimond N., Drusch F., Jacques N., Billiot F., Laplanche A., Chauchereau A., Lacroix L., Planchard D. (2011). A direct comparison of CellSearch and ISET for circulating tumour-cell detection in patients with metastatic carcinomas. Br. J. Cancer.

[34] Desitter I., Guerrouahen B.S., Benali-Furet N., Wechsler J., Janne P.A., Kuang Y., Yanagita M., Wang L., Berkowitz J.A., Distel R.J. (2011). A new device for rapid isolation by size and characterization of rare circulating tumor cells. Anticancer Res..

[35] Freidin M.B., Tay A., Freydina D.V., Chudasama D., Nicholson A.G., Rice A., Anikin V., Lim E. (2014). An assessment of diagnostic performance of a filter-based antibody-independent peripheral blood circulating tumour cell capture paired with cytomorphologic criteria for the diagnosis of cancer. Lung Cancer.

[36] Khoo B.L., Warkiani M.E., Tan D.S., Bhagat A.A., Irwin D., Lau D.P., Lim A.S., Lim K.H., Krisna S.S., Lim W.T. (2014). Clinical validation of an ultra high-throughput spiral microfluidics for the detection and enrichment of viable circulating tumor cells. PLoS ONE.

[37] Lee Y., Guan G., Bhagat A.A. (2018). ClearCell FX, a label-free microfluidics technology for enrichment of viable circulating tumor cells. Cytometry.

[38] Sollier E., Go D.E., Che J., Gossett D.R., O’Byrne S., Weaver W.M., Kummer N., Rettig M., Goldman J., Nickols N. (2014). Size-selective collection of circulating tumor cells using Vortex technology. Lab Chip.

[39] Rosenberg R., Gertler R., Friederichs J., Fuehrer K., Dahm M., Phelps R., Thorban S., Nekarda H., Siewert J.R. (2002). Comparison of two density gradient centrifugation systems for the enrichment of disseminated tumor cells in blood. Cytometry.

[40] Campton D.E., Ramirez A.B., Nordberg J.J., Drovetto N., Clein A.C., Varshavskaya P., Friemel B.H., Quarre S., Breman A., Dorschner M. (2015). High-recovery visual identification and single-cell retrieval of circulating tumor cells for genomic analysis using a dual-technology platform integrated with automated immunofluorescence staining. BMC Cancer.

[41] Saucedo-Zeni N., Mewes S., Niestroj R., Gasiorowski L., Murawa D., Nowaczyk P., Tomasi T., Weber E., Dworacki G., Morgenthaler N.G. (2012). A novel method for the in vivo isolation of circulating tumor cells from peripheral blood of cancer patients using a functionalized and structured medical wire. Int. J. Oncol..

[42] Gorges T.M., Penkalla N., Schalk T., Joosse S.A., Riethdorf S., Tucholski J., Lucke K., Wikman H., Jackson S., Brychta N. (2016). Enumeration and Molecular Characterization of Tumor Cells in Lung Cancer Patients Using a Novel In Vivo Device for Capturing Circulating Tumor Cells. Clin. Cancer Res..

[43] Wang D., Ge C., Liang W., Yang Q., Liu Q., Ma W., Shi L., Wu H., Zhang Y., Wu Z. (2020). In Vivo Enrichment and Elimination of Circulating Tumor Cells by Using a Black Phosphorus and Antibody Functionalized Intravenous Catheter. Adv. Sci..

[44] Kim T.H., Wang Y., Oliver C.R., Thamm D.H., Cooling L., Paoletti C., Smith K.J., Nagrath S., Hayes D.F. (2019). A temporary indwelling intravascular aphaeretic system for in vivo enrichment of circulating tumor cells. Nat. Commun..

[45] Vermesh O., Aalipour A., Ge T.J., Saenz Y., Guo Y., Alam I.S., Park S.M., Adelson C.N., Mitsutake Y., Vilches-Moure J. (2018). An intravascular magnetic wire for the high-throughput retrieval of circulating tumour cells in vivo. Nat. Biomed. Eng..

[46] Belotti Y., Lim C.T. (2021). Microfluidics for Liquid Biopsies: Recent Advances, Current Challenges, and Future Directions. Anal. Chem..

[47] Wei X., Chen K., Guo S., Liu W., Zhao X.Z. (2021). Emerging microfluidic technologies for the detection of circulating tumor cells and fetal nucleated red blood cells. ACS Appl. Bio Mater..

[48] Cristofanilli M., Hayes D.F., Budd G.T., Ellis M.J., Stopeck A., Reuben J.M., Doyle G.V., Matera J., Allard W.J., Miller M.C. (2005). Circulating tumor cells: A novel prognostic factor for newly diagnosed metastatic breast cancer. J. Clin. Oncol..

[49] Hayes D.F., Cristofanilli M., Budd G.T., Ellis M.J., Stopeck A., Miller M.C., Matera J., Allard W.J., Doyle G.V., Terstappen L.W. (2006). Circulating tumor cells at each follow-up time point during therapy of metastatic breast cancer patients predict progression-free and overall survival. Clin. Cancer Res..

[50] Muller V., Hayes D.F., Pantel K. (2006). Recent translational research: Circulating tumor cells in breast cancer patients. Breast Cancer Res..

[51] Cohen S.J., Punt C.J., Iannotti N., Saidman B.H., Sabbath K.D., Gabrail N.Y., Picus J., Morse M.A., Mitchell E., Miller M.C. (2009). Prognostic significance of circulating tumor cells in patients with metastatic colorectal cancer. Ann. Oncol..

[52] de Bono J.S., Scher H.I., Montgomery R.B., Parker C., Miller M.C., Tissing H., Doyle G.V., Terstappen L.W., Pienta K.J., Raghavan D. (2008). Circulating tumor cells predict survival benefit from treatment in metastatic castration-resistant prostate cancer. Clin. Cancer Res..

[53] Battula V.L., Evans K.W., Hollier B.G., Shi Y., Marini F.C., Ayyanan A., Wang R.Y., Brisken C., Guerra R., Andreeff M. (2010). Epithelial-mesenchymal transition-derived cells exhibit multilineage differentiation potential similar to mesenchymal stem cells. Stem Cells.

[54] Chen J., Yu L., Camacho J.L.C., Chai Y., Li D., Li Y., Liu H., Ou L., Li W., Haag R. (2019). Biospecific monolayer coating for multivalent capture of circulating tumor cells with high sensitivity. Adv. Funct. Mater..

[55] Lu N.N., Xie M., Wang J., Lv S.W., Yi J.S., Dong W.G., Huang W.H. (2015). Biotin-triggered decomposable immunomagnetic beads for capture and release of circulating tumor cells. ACS Appl. Mater. Interfaces.

[56] Mishra A., Dubash T.D., Edd J.F., Jewett M.K., Garre S.G., Karabacak N.M., Rabe D.C., Mutlu B.R., Walsh J.R., Kapur R. (2020). Ultrahigh-throughput magnetic sorting of large blood volumes for epitope-agnostic isolation of circulating tumor cells. Proc. Natl. Acad. Sci. USA.

[57] Kim J., Cho H., Han S.I., Han K.H. (2016). Single-cell isolation of circulating tumor cells from whole blood by lateral magnetophoretic microseparation and microfluidic dispensing. Anal Chem..

[58] Tang M., Wen C.Y., Wu L.L., Hong S.L., Hu J., Xu C.M., Pang D.W., Zhang Z.L. (2016). A Chip assisted immunomagnetic separation system for the efficient capture and in situ identification of circulating tumor cells. Lab Chip.

[59] Qin X., Park S., Duffy S.P., Matthews K., Ang R.R., Todenhofer T., Abdi H., Azad A., Bazov J., Chi K.N. (2015). Size and deformability based separation of circulating tumor cells from castrate resistant prostate cancer patients using resettable cell traps. Lab Chip.

[60] Kim T.H., Lim M., Park J., Oh J.M., Kim H., Jeong H., Lee S.J., Park H.C., Jung S., Kim B.C. (2017). FAST: Size-Selective, Clog-Free Isolation of Rare Cancer Cells from Whole Blood at a Liquid-Liquid Interface. Anal. Chem..

[61] Sarioglu A.F., Aceto N., Kojic N., Donaldson M.C., Zeinali M., Hamza B., Engstrom A., Zhu H., Sundaresan T.K., Miyamoto D.T. (2015). A microfluidic device for label-free, physical capture of circulating tumor cell clusters. Nat. Methods.

[62] Ahmed M.G., Abate M.F., Song Y., Zhu Z., Yan F., Xu Y., Wang X., Li Q., Yang C. (2017). Isolation, Detection, and Antigen-Based Profiling of Circulating Tumor Cells Using a Size-Dictated Immunocapture Chip. Angew. Chem. Int. Ed. Engl..

[63] Sequist L.V., Nagrath S., Toner M., Haber D.A., Lynch T.J. (2009). The CTC-chip: An exciting new tool to detect circulating tumor cells in lung cancer patients. J. Thorac. Oncol..

[64] Lin E., Rivera-Baez L., Fouladdel S., Yoon H.J., Guthrie S., Wieger J., Deol Y., Keller E., Sahai V., Simeone D.M. (2017). High-Throughput Microfluidic Labyrinth for the Label-free Isolation of Circulating Tumor Cells. Cell Syst..

[65] Lemaire C.A., Liu S.Z., Wilkerson C.L., Ramani V.C., Barzanian N.A., Huang K.W., Che J., Chiu M.W., Vuppalapaty M., Dimmick A.M. (2018). Fast and Label-Free Isolation of Circulating Tumor Cells from Blood: From a Research Microfluidic Platform to an Automated Fluidic Instrument, VTX-1 Liquid Biopsy System. SLAS Technol..

[66] Zheng S., Lin H.K., Lu B., Williams A., Datar R., Cote R.J., Tai Y.C. (2011). 3D microfilter device for viable circulating tumor cell (CTC) enrichment from blood. Biomed. Microdevices.

[67] Hosokawa M., Yoshikawa T., Negishi R., Yoshino T., Koh Y., Kenmotsu H., Naito T., Takahashi T., Yamamoto N., Kikuhara Y. (2013). Microcavity array system for size-based enrichment of circulating tumor cells from the blood of patients with small-cell lung cancer. Anal. Chem..

[68] Di Carlo D. (2009). Inertial microfluidics. Lab Chip.

[69] Di Carlo D., Irimia D., Tompkins R.G., Toner M. (2007). Continuous inertial focusing, ordering, and separation of particles in microchannels. Proc. Natl. Acad. Sci. USA.

[70] Abdulla A., Liu W., Gholamipour-Shirazi A., Sun J., Ding X. (2018). High-Throughput Isolation of Circulating Tumor Cells Using Cascaded Inertial Focusing Microfluidic Channel. Anal. Chem..

[71] Kuntaegowdanahalli S.S., Bhagat A.A., Kumar G., Papautsky I. (2009). Inertial microfluidics for continuous particle separation in spiral microchannels. Lab Chip.

[72] Nivedita N., Garg N., Lee A.P., Papautsky I. (2017). A high throughput microfluidic platform for size-selective enrichment of cell populations in tissue and blood samples. Analyst.

[73] Huang L.R., Cox E.C., Austin R.H., Sturm J.C. (2004). Continuous particle separation through deterministic lateral displacement. Science.

[74] McGrath J., Jimenez M., Bridle H. (2014). Deterministic lateral displacement for particle separation: A review. Lab Chip.

[75] Gascoyne P.R., Shim S. (2014). Isolation of circulating tumor cells by dielectrophoresis. Cancers.

[76] Cappelletti V., Verzoni E., Ratta R., Vismara M., Silvestri M., Montone R., Miodini P., Reduzzi C., Claps M., Sepe P. (2020). Analysis of Single Circulating Tumor Cells in Renal Cell Carcinoma Reveals Phenotypic Heterogeneity and Genomic Alterations Related to Progression. Int. J. Mol. Sci..

[77] Bidard F.C., Mathiot C., Delaloge S., Brain E., Giachetti S., de Cremoux P., Marty M., Pierga J.Y. (2010). Single circulating tumor cell detection and overall survival in nonmetastatic breast cancer. Ann. Oncol..

[78] Bidard F.C., Belin L., Delaloge S., Lerebours F., Ngo C., Reyal F., Alran S., Giacchetti S., Marty M., Lebofsky R. (2013). Time-Dependent Prognostic Impact of Circulating Tumor Cells Detection in Non-Metastatic Breast Cancer: 70-Month Analysis of the REMAGUS02 Study. Int. J. Breast Cancer.

[79] Giacchetti S., Hamy A.S., Delaloge S., Brain E., Berger F., Sigal-Zafrani B., Mathieu M.C., Bertheau P., Guinebretiere J.M., Saghatchian M. (2017). Long-term outcome of the REMAGUS 02 trial, a multicenter randomised phase II trial in locally advanced breast cancer patients treated with neoadjuvant chemotherapy with or without celecoxib or trastuzumab according to HER2 status. Eur. J. Cancer.

[80] Sparano J., O’Neill A., Alpaugh K., Wolff A.C., Northfelt D.W., Dang C.T., Sledge G.W., Miller K.D. (2018). Association of Circulating Tumor Cells with Late Recurrence of Estrogen Receptor-Positive Breast Cancer: A Secondary Analysis of a Randomized Clinical Trial. JAMA Oncol..

[81] Radovich M., Jiang G., Hancock B.A., Chitambar C., Nanda R., Falkson C., Lynce F.C., Gallagher C., Isaacs C., Blaya M. (2020). Association of Circulating Tumor DNA and Circulating Tumor Cells After Neoadjuvant Chemotherapy with Disease Recurrence in Patients with Triple-Negative Breast Cancer: Preplanned Secondary Analysis of the BRE12-158 Randomized Clinical Trial. JAMA Oncol..

[82] Heidrich I., Ackar L., Mossahebi Mohammadi P., Pantel K. (2020). Liquid biopsies: Potential and challenges. Int. J. Cancer.

[83] Kelley S.O., Pantel K. (2020). A New Era in Liquid Biopsy: From Genotype to Phenotype. Clin. Chem..

[84] Krebs M.G., Hou J.M., Ward T.H., Blackhall F.H., Dive C. (2010). Circulating tumour cells: Their utility in cancer management and predicting outcomes. Ther Adv. Med. Oncol..

[85] Cristofanilli M., Budd G.T., Ellis M.J., Stopeck A., Matera J., Miller M.C., Reuben J.M., Doyle G.V., Allard W.J., Terstappen L.W. (2004). Circulating tumor cells, disease progression, and survival in metastatic breast cancer. N. Engl. J. Med..

[86] Larsson A.M., Jansson S., Bendahl P.O., Levin Tykjaer Jorgensen C., Loman N., Graffman C., Lundgren L., Aaltonen K., Ryden L. (2018). Longitudinal enumeration and cluster evaluation of circulating tumor cells improve prognostication for patients with newly diagnosed metastatic breast cancer in a prospective observational trial. Breast Cancer Res..

[87] Cohen S.J., Punt C.J., Iannotti N., Saidman B.H., Sabbath K.D., Gabrail N.Y., Picus J., Morse M., Mitchell E., Miller M.C. (2008). Relationship of circulating tumor cells to tumor response, progression-free survival, and overall survival in patients with metastatic colorectal cancer. J. Clin. Oncol..

[88] Basso U., Facchinetti A., Rossi E., Maruzzo M., Conteduca V., Aieta M., Massari F., Fraccon A.P., Mucciarini C., Sava T. (2021). Prognostic Role of Circulating Tumor Cells in Metastatic Renal Cell Carcinoma: A Large, Multicenter, Prospective Trial. Oncologist.

[89] Cristofanilli M., Pierga J.Y., Reuben J., Rademaker A., Davis A.A., Peeters D.J., Fehm T., Nole F., Gisbert-Criado R., Mavroudis D. (2019). The clinical use of circulating tumor cells (CTCs) enumeration for staging of metastatic breast cancer (MBC): International expert consensus paper. Crit. Rev. Oncol. Hematol..

[90] Smerage J.B., Barlow W.E., Hortobagyi G.N., Winer E.P., Leyland-Jones B., Srkalovic G., Tejwani S., Schott A.F., O’Rourke M.A., Lew D.L. (2014). Circulating tumor cells and response to chemotherapy in metastatic breast cancer: SWOG S0500. J. Clin. Oncol..

[91] Pierga J.Y., Bidard F.C., Cropet C., Tresca P., Dalenc F., Romieu G., Campone M., Mahier Ait-Oukhatar C., Le Rhun E., Goncalves A. (2013). Circulating tumor cells and brain metastasis outcome in patients with HER2-positive breast cancer: The LANDSCAPE trial. Ann. Oncol..

[92] Giuliano M., Shaikh A., Lo H.C., Arpino G., De Placido S., Zhang X.H., Cristofanilli M., Schiff R., Trivedi M.V. (2018). Perspective on Circulating Tumor Cell Clusters: Why It Takes a Village to Metastasize. Cancer Res..

[93] Reduzzi C., Di Cosimo S., Gerratana L., Motta R., Martinetti A., Vingiani A., D’Amico P., Zhang Y., Vismara M., Depretto C. (2021). Circulating Tumor Cell Clusters Are Frequently Detected in Women with Early-Stage Breast Cancer. Cancers.

[94] Murlidhar V., Reddy R.M., Fouladdel S., Zhao L., Ishikawa M.K., Grabauskiene S., Zhang Z., Lin J., Chang A.C., Carrott P. (2017). Poor Prognosis Indicated by Venous Circulating Tumor Cell Clusters in Early-Stage Lung Cancers. Cancer Res..

[95] Jansson S., Bendahl P.O., Larsson A.M., Aaltonen K.E., Ryden L. (2016). Prognostic impact of circulating tumor cell apoptosis and clusters in serial blood samples from patients with metastatic breast cancer in a prospective observational cohort. BMC Cancer.

[96] Hou J.M., Krebs M.G., Lancashire L., Sloane R., Backen A., Swain R.K., Priest L.J., Greystoke A., Zhou C., Morris K. (2012). Clinical significance and molecular characteristics of circulating tumor cells and circulating tumor microemboli in patients with small-cell lung cancer. J. Clin. Oncol..

[97] Wang C., Mu Z., Chervoneva I., Austin L., Ye Z., Rossi G., Palazzo J.P., Sun C., Abu-Khalaf M., Myers R.E. (2017). Longitudinally collected CTCs and CTC-clusters and clinical outcomes of metastatic breast cancer. Breast Cancer Res. Treat..

[98] Au S.H., Edd J., Stoddard A.E., Wong K.H.K., Fachin F., Maheswaran S., Haber D.A., Stott S.L., Kapur R., Toner M. (2017). Microfluidic Isolation of Circulating Tumor Cell Clusters by Size and Asymmetry. Sci. Rep..

[99] Cheng S.B., Xie M., Chen Y., Xiong J., Liu Y., Chen Z., Guo S., Shu Y., Wang M., Yuan B.F. (2017). Three-Dimensional Scaffold Chip with Thermosensitive Coating for Capture and Reversible Release of Individual and Cluster of Circulating Tumor Cells. Anal. Chem..

[100] Kulasinghe A., Zhou J., Kenny L., Papautsky I., Punyadeera C. (2019). Capture of Circulating Tumour Cell Clusters Using Straight Microfluidic Chips. Cancers.

[101] Wang C., Zhang Z., Chong W., Luo R., Myers R.E., Gu J., Lin J., Wei Q., Li B., Rebbeck T.R. (2021). Improved Prognostic Stratification Using Circulating Tumor Cell Clusters in Patients with Metastatic Castration-Resistant Prostate Cancer. Cancers.

[102] Krol I., Schwab F.D., Carbone R., Ritter M., Picocci S., De Marni M.L., Stepien G., Franchi G.M., Zanardi A., Rissoglio M.D. (2021). Detection of clustered circulating tumour cells in early breast cancer. Br. J. Cancer.

[103] Costa C., Muinelo-Romay L., Cebey-Lopez V., Pereira-Veiga T., Martinez-Pena I., Abreu M., Abalo A., Lago-Leston R.M., Abuin C., Palacios P. (2020). Analysis of a Real-World Cohort of Metastatic Breast Cancer Patients Shows Circulating Tumor Cell Clusters (CTC-clusters) as Predictors of Patient Outcomes. Cancers.

[104] Paoletti C., Miao J., Dolce E.M., Darga E.P., Repollet M.I., Doyle G.V., Gralow J.R., Hortobagyi G.N., Smerage J.B., Barlow W.E. (2019). Circulating Tumor Cell Clusters in Patients with Metastatic Breast Cancer: A SWOG S0500 Translational Medicine Study. Clin. Cancer Res..

[105] Ried K., Eng P., Sali A. (2017). Screening of Circulating Tumor Cells Allow Early Detection of Cancer and Monitoring of Treatment Effectiveness: An Observational Study. Asian Pac. J. Cancer Prev..

[106] Nimgaonkar A., Segurado O., Tsai W.S., Pang S.T., Hou M.F., Chang Y., Watson D., Chang Y.H., Lin P.H., Wu J.C. (2018). A novel circulating utmor cell blood test for early detection of colorectal, prostate and breast cancers: Result from 709 samples. J. Clin. Oncol..

[107] Ludwig J.A., Weinstein J.N. (2005). Biomarkers in Cancer Staging, Prognosis and Treatment Selection. Nat. Rev. Cancer.

[108] Sauter E.R. (2017). Reliable Biomarkers to Identify New and Recurrent Cancer. Eur. J. Breat Health.

[109] Herrmann K., Walch A., Balluff B., Tanzer M., Hofler H., Krause B.J., Schwaiger M., Friess H., Schmid R.M., Ebert M.P.A. (2009). Proteomic and metabolic prediction of response to therapy in gastrointestinal cancers. Nat. Rev. Gastroenterol. Hepatol..

[110] Pantel K., Alix-Panabieres C. (2019). Liquid biopsy and minimal residual disease-latest advances and implications for cure. Nat. Rev. Clin. Oncol.

[111] Goodman C.R., Seagle B.L., Friedl T.W.P., Rack B., Lato K., Fink V., Cristofanilli M., Donnelly E.D., Janni W., Shahabi S. (2018). Association of Circulating Tumor Cell Status With Benefit of Radiotherapy and Survival in Early-Stage Breast Cancer. JAMA Oncol..

[112] Bidard F.C., Mathiot C., Degeorges A., Etienne-Grimaldi M.C., Delva R., Pivot X., Veyret C., Bergougnoux L., de Cremoux P., Milano G. (2010). Clinical value of circulating endothelial cells and circulating tumor cells in metastatic breast cancer patients treated first line with bevacizumab and chemotherapy. Ann. Oncol..

[113] Giuliano M., Giordano A., Jackson S., Hess K.R., De Giorgi U., Mego M., Handy B.C., Ueno N.T., Alvarez R.H., De Laurentiis M. (2011). Circulating tumor cells as prognostic and predictive markers in metastatic breast cancer patients receiving first-line systemic treatment. Breast Cancer Res..

[114] Bidard F.C., Jacot W., Kiavue N., Dureau S., Kadi A., Brain E., Bachelot T., Bourgeois H., Goncalves A., Ladoire S. (2020). Efficacy of Circulating Tumor Cell Count-Driven vs Clinician-Driven First-line Therapy Choice in Hormone Receptor-Positive, ERBB2-Negative Metastatic Breast Cancer: The STIC CTC Randomized Clinical Trial. JAMA Oncol..

[115] Punnoose E.A., Atwal S., Liu W., Raja R., Fine B.M., Hughes B.G., Hicks R.J., Hampton G.M., Amler L.C., Pirzkall A. (2012). Evaluation of circulating tumor cells and circulating tumor DNA in non-small cell lung cancer: Association with clinical endpoints in a phase II clinical trial of pertuzumab and erlotinib. Clin. Cancer Res..

[116] Hiltermann T.J.N., Pore M.M., van den Berg A., Timens W., Boezen H.M., Liesker J.J.W., Schouwink J.H., Wijnands W.J.A., Kerner G., Kruyt F.A.E. (2012). Circulating tumor cells in small-cell lung cancer: A predictive and prognostic factor. Ann. Oncol..

[117] Fu L., Liu F., Fu H., Liu L., Yuan S., Gao Y., Fu Z., Yu J. (2014). Circulating tumor cells correlate with recurrence in stage III small-cell lung cancer after systemic chemoradiotherapy and prophylactic cranial irradiation. Jpn. J. Clin. Oncol..

[118] Fu L., Zhu Y., Jing W., Guo D., Kong L., Yu J. (2018). Incorporation of circulating tumor cells and whole-body metabolic tumor volume of (18)F-FDG PET/CT improves prediction of outcome in IIIB stage small-cell lung cancer. Chin. J. Cancer Res..

[119] Heller G., McCormack R., Kheoh T., Molina A., Smith M.R., Dreicer R., Saad F., de Wit R., Aftab D.T., Hirmand M. (2018). Circulating Tumor Cell Number as a Response Measure of Prolonged Survival for Metastatic Castration-Resistant Prostate Cancer: A Comparison With Prostate-Specific Antigen Across Five Randomized Phase III Clinical Trials. J. Clin. Oncol..

[120] Goldkorn A., Tangen C., Plets M., Morrison G.J., Cunha A., Xu T., Pinski J.K., Ingles S.A., Triche T., Harzstark A.L. (2021). Baseline Circulating Tumor Cell Count as a Prognostic Marker of PSA Response and Disease Progression in Metastatic Castrate-Sensitive Prostate Cancer (SWOG S1216). Clin. Cancer Res..

[121] Yu M., Bardia A., Aceto N., Bersani F., Madden M.W., Donaldson M.C., Desai R., Zhu H., Comaills V., Zheng Z. (2014). Ex Vivo culture of circulating breast tumor cells for individualized testing of drug susceptibility. Science.

[122] Lallo A., Schenk M.W., Frese K.K., Blackhall F., Dive C. (2017). Circulating tumor cells and CDx models as a tool for preclinical drug development. Transl. Lung Cancer Res..

[123] Conteduca V., Ku S.Y., Fernandez L., Rodriquez A.D., Lee J., Jendrisak A., Slade M., Gilbertson C., Manohar J., Sigouros M. (2021). Circulating tumor cell heterogeneity in neuroendocrine prostate cancer by single cell copy number analysis. NPJ Precis. Oncol..

[124] Kong S.L., Liu X., Tan S.J., Tai J.A., Phua L.Y., Poh H.M., Yeo T., Chua Y.W., Haw Y.X., Ling W.H. (2021). Complementary Sequential Circulating Tumor Cell (CTC) and Cell-Free Tumor DNA (ctDNA) Profiling Reveals Metastatic Heterogeneity and Genomic Changes in Lung Cancer and Breast Cancer. Front. Oncol..

[125] Genna A., Vanwynsberghe A.M., Villard A.V., Pottier C., Ancel J., Polette M., Gilles C. (2020). EMT-Associated Heterogeneity in Circulating Tumor Cells: Sticky Friends on the Road to Metastasis. Cancers.

[126] Yu M., Bardia A., Wittner B.S., Stott S.L., Smas M.E., Ting D.T., Isakoff S.J., Ciciliano J.C., Wells M.N., Shah A.M. (2013). Circulating breast tumor cells exhibit dynamic changes in epithelial and mesenchymal composition. Science.

[127] Koch C., Kuske A., Joosse S.A., Yigit G., Sflomos G., Thanler S., Smit D.J., Werner S., Borgmann K., Gartner S. (2020). Characterization of circulating breast cancer cells with tumorigenic and metastatic capacity. EMBO Mol. Med..

[128] Castro-Giner F., Aceto N. (2020). Tracking cancer progression: From circulating tumor cells to metastasis. Genome Med..

[129] Franses J.W., Philipp J., Missios P., Bhan I., Liu A., Yashaswini C., Tai E., Zhu H., Ligorio M., Nicholson B. (2020). Pancreatic circulating tumor cell profiling identifies LIN28B as a metastasis driver and drug target. Nat. Commun..

[130] Pailler E., Faugeroux V., Oulhen M., Mezquita L., Laporte M., Honore A., Lecluse Y., queffelec P., NgoCamus M., Nicorta C. (2019). Acquired Resistance Mutations to ALK inhibitors Identified by Single Circulating Tumor Cell Sequencing in ALK-Rearranged Non-Small-Cell Lung Cancer. Clin. Can. Res..

[131] Medford A.J., Dubash T.D., Juric D., Spring L., Niemierko A., Vidula N., Peppercorn J., Isakoff S., Reeves B.A., LiCausi J.A. (2019). Blood-based monitoring identifies acquired and targetable driver mutations in endocrine-resistant metastatic breasr cancer. Npj Precis. Oncol..

[132] Wang J., Xu B. (2019). Targeted therapeutic options and future perspectives for HER2-positive breast cancer. Signal Transduct. Target. Ther..

[133] Beije N., Onstenk W., Kraan J., Sieuwerts A.M., Hamberg P., Dirix L.Y., Brouwer A., de Jongh F.E., Jager A., Seynaeve C.M. (2016). Prognostic Impact of HER2 and ER Status of Circulating Tumor Cells in Metastatic Breast Cancer Patients with a HER2-Negative Primary Tumor. Neoplasia.

[134] Fehm T., Muller V., Aktas B., Janni W., Schneeweiss A., Stickeler E., Lattrich C., Lohberg C.R., Solomayer E., Rack B. (2010). HER2 status of circulating tumor cells in patients with metastatic breast cancer: A prospective, multicenter trial. Breast Cancer Res. Treat..

[135] Liu Y., Liu Q., Wang T., Bian L., Zhang S., Hu H., Li S., Hu Z., Wu S., Liu B. (2013). Circulating tumor cells in HER2-positive metastatic breast cancer patients: A valuable prognostic and predictive biomarker. BMC Cancer.

[136] Zhang S., Li L., Wang T., Bian L., Hu H., Xu C., Liu B., Liu Y., Cristofanilli M., Jiang Z. (2016). Real-time HER2 status detected on circulating tumor cells predicts different outcomes of anti-HER2 therapy in histologically HER2-positive metastatic breast cancer patients. BMC Cancer.

[137] Jacot W., Cottu P., Berger F., Dubot C., Venat-Bouvet L., Lortholary A., Bourgeois H., Bollet M., Servent V., Luporsi E. (2019). Actionability of HER2-amplified circulating tumor cells in HER2-negative metastatic breast cancer: The CirCe T-DM1 trial. Breast Cancer Res..

[138] Ignatiadis M., Litiere S., Rothe F., Riethdorf S., Proudhon C., Fehm T., Aalders K., Forstbauer H., Fasching P.A., Brain E. (2018). Trastuzumab versus observation for HER2 nonamplified early breast cancer with circulating tumor cells (EORTC 90091-10093, BIG 1-12, Treat CTC): A randomized phase II trial. Ann. Oncol..

[139] Riethdorf S., Muller V., Zhang L., Rau T., Loibl S., Komor M., Roller M., Huober J., Fehm T., Schrader I. (2010). Detection and HER2 expression of circulating tumor cells: Prospective monitoring in breast cancer patients treated in the neoadjuvant GeparQuattro trial. Clin. Cancer Res..

[140] Pobiruchin M., Bochum S., Martens U.M., Kieser M., Schramm W. (2016). Transition probabilities of HER2-positive and HER2-negative breast cancer patients treated with Trastuzumab obtained from a clinical cancer registry dataset. Data Brief..

[141] Deutsch T.M., Riethdorf S., Fremd C., Feisst M., Nees J., Fischer C., Hartkopf A.D., Pantel K., Trumpp A., Schutz F. (2020). HER2-targeted therapy influences CTC status in metastatic breast cancer. Breast Cancer Res. Treat..

[142] Serrano M.J., Alvarez-Cubero M.J., De Miguel Perez D., Rodriguez-Martinez A., Gonzalez-Herrera L., Robles-Fernandez I., Hernandez J.E., Puche J.L.G., Lorente J.A. (2017). Significance of EGFR Expression in Circulating Tumor Cells. Adv. Exp. Med. Biol..

[143] Maheswaran S., Sequist L.V., Nagrath S., Ulkus L., Brannigan B., Collura C.V., Inserra E., Diederichs S., Iafrate A.J., Bell D.W. (2008). Detection of mutations in EGFR in circulating lung-cancer cells. N. Engl. J. Med..

[144] Godin-Heymann N., Ulkus L., Brannigan B.W., McDermott U., Lamb J., Maheswaran S., Settleman J., Haber D.A. (2008). The T790M “gatekeeper” mutation in EGFR mediates resistance to low concentrations of an irreversible EGFR inhibitor. Mol. Cancer Ther..

[145] Wang D.D., Ma L., Wong M.P., Lee V.H., Yan H. (2015). Contribution of EGFR and ErbB-3 Heterodimerization to the EGFR Mutation-Induced Gefitinib- and Erlotinib-Resistance in Non-Small-Cell Lung Carcinoma Treatments. PLoS ONE.

[146] Kuboki Y., Matsusaka S., Minowa S., Shibata H., Suenaga M., Shinozaki E., Mizunuma N., Ueno M., Yamaguchi T., Hatake K. (2013). Circulating tumor cell (CTC) count and epithelial growth factor receptor expression on CTCs as biomarkers for cetuximab efficacy in advanced colorectal cancer. Anticancer Res..

[147] Okegawa T., Itaya N., Hara H., Tambo M., Nutahara K. (2016). Epidermal Growth Factor Receptor Status in Circulating Tumor Cells as a Predictive Biomarker of Sensitivity in Castration-Resistant Prostate Cancer Patients Treated with Docetaxel Chemotherapy. Int. J. Mol. Sci..

[148] Serrano M.J., Ortega F.G., Alvarez-Cubero M.J., Nadal R., Sanchez-Rovira P., Salido M., Rodriguez M., Garcia-Puche J.L., Delgado-Rodriguez M., Sole F. (2014). EMT and EGFR in CTCs cytokeratin negative non-metastatic breast cancer. Oncotarget.

[149] Braun A.C., de Mello C.A.L., Corassa M., Abdallah E.A., Urvanegia A.C., Alves V.S., Flores B., Diaz M., Nicolau U.R., Silva V.S.E. (2018). EGFR expression in circulating tumor cells from high-grade metastatic soft tissue sarcomas. Cancer Biol. Ther..

[150] Kallergi G., Agelaki S., Kalykaki A., Stournaras C., Mavroudis D., Georgoulias V. (2008). Phosphorylated EGFR and PI3K/Akt signaling kinases are expressed in circulating tumor cells of breast cancer patinets. Breast Cancer Res..

[151] Meador C.B., Jin H., de Stanchina E., Nebhan C.A., Pirazzoli V., Wang L., Lu P., Vuong H., Hutchinson K.E., Jia P. (2015). Optimizing the sequence of anti-EGFR-targeted therapy in EGFR-mutant lung cancer. Mol. Cancer Ther..

[152] Nguyen K.S., Kobayashi S., Costa D.B. (2009). Acquired resistance to epidermal growth factor receptor tyrosine kinase inhibitors in non-small-cell lung cancers dependent on the epidermal growth factor receptor pathway. Clin. Lung Cancer.

[153] Matsuo N., Azuma K., Sakai K., Hattori S., Kawahara A., Ishii H., Tokito T., Kinoshita T., Yamada K., Nishio K. (2016). Association of EGFR Exon 19 Deletion and EGFR-TKI Treatment Duration with Frequency of T790M Mutation in EGFR-Mutant Lung Cancer Patients. Sci. Rep..

[154] Gao W., He J., Jin S.D., Xu J., Yu T.F., Wang W., Zhu Q., Dai H., Wu H., Liu Y.Q. (2019). Association of Initial Epidermal Growth Factor Receptor Tyrosine Kinase Inhibitors Treatment and EGFR Exon 19 Deletion with Frequency of The T790M Mutation in Non-Small Cell Lung Cancer Patients After Resistance To First-Line Epidermal Growth Factor Receptor Tyrosine Kinase Inhibitors. Onco. Targets Ther..

[155] Wang Z.F., Ren S.X., Li W., Gao G.H. (2018). Frequency of the acquired resistant mutation T790 M in non-small cell lung cancer patients with active exon 19Del and exon 21 L858R: A systematic review and meta-analysis. BMC Cancer.

[156] Sundaresan T.K., Sequist L.V., Heymach J.V., Riely G.J., Janne P.A., Koch W.H., Sullivan J.P., Fox D.B., Maher R., Muzikansky A. (2016). Detection of T790M, the Acquired Resistance EGFR Mutation, by Tumor Biopsy versus Noninvasive Blood-Based Analyses. Clin. Cancer Res..

[157] Huang C.W., Chen Y.T., Tsai H.L., Yeh Y.S., Su W.C., Ma C.J., Tsai T.N., Wang J.Y. (2017). EGFR expression in patients with stage III colorectal cancer after adjuvant chemotherapy and on cancer cell function. Oncotarget.

[158] Liu H.E., Vuppalapaty M., Wilkerson C., Renier C., Chiu M., Lemaire C., Che J., Matsumoto M., Carroll J., Crouse S. (2020). Detection of EGFR Mutations in cfDNA and CTCs, and Comparison to Tumor Tissue in Non-Small-Cell-Lung-Cancer (NSCLC) Patients. Front. Oncol..

[159] Schweizer M.T., Antonarakis E.S. (2015). Liquid biopsy: Clues on prostate cancer drug resistance. Sci. Transl. Med..

[160] Antonarakis E.S. (2015). Predicting treatment response in castration-resistant prostate cancer: Could androgen receptor variant-7 hold the key?. Expert Rev. Anticancer Ther..

[161] Antonarakis E.S., Lu C., Wang H., Luber B., Nakazawa M., Roeser J.C., Chen Y., Mohammad T.A., Chen Y., Fedor H.L. (2014). AR-V7 and resistance to enzalutamide and abiraterone in prostate cancer. N. Engl. J. Med..

[162] Antonarakis E.S., Lu C., Luber B., Wang H., Chen Y., Nakazawa M., Nadal R., Paller C.J., Denmeade S.R., Carducci M.A. (2015). Androgen Receptor Splice Variant 7 and Efficacy of Taxane Chemotherapy in Patients With Metastatic Castration-Resistant Prostate Cancer. JAMA Oncol..

[163] Guo Z., Yang X., Sun F., Jiang R., Linn D.E., Chen H., Chen H., Kong X., Melamed J., Tepper C.G. (2009). A novel androgen receptor splice variant is up-regulated during prostate cancer progression and promotes androgen depletion-resistant growth. Cancer Res..

[164] Li Y., Chan S.C., Brand L.J., Hwang T.H., Silverstein K.A., Dehm S.M. (2013). Androgen receptor splice variants mediate enzalutamide resistance in castration-resistant prostate cancer cell lines. Cancer Res..

[165] Scher H.I., Lu D., Schreiber N.A., Louw J., Graf R.P., Vargas H.A., Johnson A., Jendrisak A., Bambury R., Danila D. (2016). Association of AR-V7 on Circulating Tumor Cells as a Treatment-Specific Biomarker with Outcomes and Survival in Castration-Resistant Prostate Cancer. JAMA Oncol..

[166] Scher H.I., Graf R.P., Schreiber N.A., Jayaram A., Winquist E., McLaughlin B., Lu D., Fleisher M., Orr S., Lowes L. (2018). Assessment of the Validity of Nuclear-Localized Androgen Receptor Splice Variant 7 in Circulating Tumor Cells as a Predictive Biomarker for Castration-Resistant Prostate Cancer. JAMA Oncol..

[167] Hille C., Gorges T.M., Riethdorf S., Mazel M., Steuber T., Amsberg G.V., Konig F., Peine S., Alix-Panabieres C., Pantel K. (2019). Detection of Androgen Receptor Variant 7 (ARV7) mRNA Levels in EpCAM-Enriched CTC Fractions for Monitoring Response to Androgen Targeting Therapies in Prostate Cancer. Cells.

[168] Zhang T., Karsh L.I., Nissenblatt M.J., Canfield S.E. (2020). Androgen Receptor Splice Variant, AR-V7, as a Biomarker of Resistance to Androgen Axis-Targeted Therapies in Advanced Prostate Cancer. Clin. Genitourin Cancer.

[169] Belderbos B.P.S., Sieuwerts A.M., Hoop E.O., Mostert B., Kraan J., Hamberg P., Van M.N., Beaufort C.M., Onstenk W., van Soest R.J. (2019). Associations between AR-V7 status in circulating tumour cells, circulating tumour cell count and survival in men with metastatic castration-resistant prostate cancer. Eur. J. Cancer.

[170] Armstrong A.J., Halabi S., Luo J., Nanus D.M., Giannakakou P., Szmulewitz R.Z., Danila D.C., Healy P., Anand M., Rothwell C.J. (2019). Prospective Multicenter Validation of Androgen Receptor Splice Variant 7 and Hormone Therapy Resistance in High-Risk Castration-Resistant Prostate Cancer: The PROPHECY Study. J. Clin. Oncol..

[171] Hench I.B., Cathomas R., Costa L., Fischer N., Gillessen S., Hench J., Hermanns T., Kremer E., Mingrone W., Mestre R.P. (2019). Analysis of AR/ARV7 Expression in Isolated Circulating Tumor Cells of Patients with Metastatic Castration-Resistant Prostate Cancer (SAKK 08/14 IMPROVE Trial). Cancers.

[172] Sakamoto S. (2019). Current status of circulating tumor cell androgen receptor splice variant-7 in metastatic castration-resistant prostate cancer. Ann. Transl. Med..

[173] Sepe P., Verzoni E., Miodini P., Claps M., Ratta R., Martinetti A., Mennitto R., Sottotetti E., Procopio G., Cappelletti V. (2019). Could Circulating Tumor Cells and ARV7 Detection Improve Clinical Decisions in Metastatic Castration-Resistant Prostate Cancer? The Istituto Nazionale dei Tumori (INT) Experience. Cancers.

[174] Engelman J.A., Zejnullahu K., Mitsudomi T., Song Y., Hyland C., Park J.O., Lindeman N., Gale C.M., Zhao X., Christensen J. (2007). MET amplification leads to gefitinib resistance in lung cancer by activating ERBB3 signaling. Science.

[175] Turke A.B., Zejnullahu K., Wu Y.L., Song Y., Dias-Santagata D., Lifshits E., Toschi L., Rogers A., Mok T., Sequist L. (2010). Preexistence and clonal selection of MET amplification in EGFR mutant NSCLC. Cancer Cell.

[176] Chen A.P., Zhang J., Liu H., Zhao S.P., Dai S.Z., Sun X.L. (2009). Association of EGFR expression with angiogenesis and chemoresistance in ovarian carcinoma. Zhonghua Zhong Liu Za Zhi.

[177] Bardelli A., Corso S., Bertotti A., Hobor S., Valtorta E., Siravegna G., Sartore-Bianchi A., Scala E., Cassingena A., Zecchin D. (2013). Amplification of the MET receptor drives resistance to anti-EGFR therapies in colorectal cancer. Cancer Discov..

[178] Liska D., Chen C.T., Bachleitner-Hofmann T., Christensen J.G., Weiser M.R. (2011). HGF rescues colorectal cancer cells from EGFR inhibition via MET activation. Clin. Cancer Res..

[179] Tada H., Takahashi H., Kawabata-Iwakawa R., Nagata Y., Uchida M., Shino M., Ida S., Mito I., Matsuyama T., Chikamatsu K. (2020). Molecular phenotypes of circulating tumor cells and efficacy of nivolumab treatment in patients with head and neck squamous cell carcinoma. Sci. Rep..

[180] Tada H., Takahashi H., Kuwabara-Yokobori Y., Shino M., Chikamatsu K. (2020). Molecular profiling of circulating tumor cells predicts clinical outcome in head and neck squamous cell carcinoma. Oral Oncol..

[181] Mondelo-Macia P., Rodriguez-Lopez C., Valina L., Aguin S., Leon-Mateos L., Garcia-Gonzalez J., Abalo A., Rapado-Gonzalez O., Suarez-Cunqueiro M., Diaz-Lagares A. (2020). Detection of MET Alterations Using Cell Free DNA and Circulating Tumor Cells from Cancer Patients. Cells.

[182] Zhang T., Boominathan R., Foulk B., Rao C., Kemeny G., Strickler J.H., Abbruzzese J.L., Harrison M.R., Hsu D.S., Healy P. (2016). Development of a Novel c-MET-Based CTC Detection Platform. Mol. Cancer Res..

[183] Wu S., Li G., Zhao X., Xiang J., Lizaso A., Ye J., Shi C., Chen L. (2020). High-level gain of mesenchymal-epithelial transition factor (MET) copy number using next-generation sequencing as a predictive biomarker for MET inhibitor efficacy. Ann. Transl. Med..

[184] Wolf J., Seto T., Han J.Y., Reguart N., Garon E.B., Groen H.J.M., Tan D.S.W., Hida T., de Jonge M., Orlov S.V. (2020). Capmatinib in MET Exon 14-Mutated or MET-Amplified Non-Small-Cell Lung Cancer. N. Engl. J. Med..

[185] Kim J., Park K.E., Jeong Y.S., Kim Y., Park H., Nam J.H., Jung K., Son W.S., Jung H.S., Lee J.H. (2020). Therapeutic Efficacy of ABN401, a Highly Potent and Selective MET Inhibitor, Based on Diagnostic Biomarker Test in MET-Addicted Cancer. Cancers.

[186] Janning M., Kobus F., Babayan A., Wikman H., Velthaus J.L., Bergmann S., Schatz S., Falk M., Berger L.A., Bottcher L.M. (2019). Determination of PD-L1 Expression in Circulating Tumor Cells of NSCLC Patients and Correlation with Response to PD-1/PD-L1 Inhibitors. Cancers.

[187] Manjunath Y., Upparahalli S.V., Avella D.M., Deroche C.B., Kimchi E.T., Staveley-O’Carroll K.F., Smith C.J., Li G., Kaifi J.T. (2019). PD-L1 Expression with Epithelial Mesenchymal Transition of Circulating Tumor Cells Is Associated with Poor Survival in Curatively Resected Non-Small Cell Lung Cancer. Cancers.

[188] Nicolazzo C., Raimondi C., Mancini M., Caponnetto S., Gradilone A., Gandini O., Mastromartino M., Del Bene G., Prete A., Longo F. (2016). Monitoring PD-L1 positive circulating tumor cells in non-small cell lung cancer patients treated with the PD-1 inhibitor Nivolumab. Sci. Rep..

[189] Adams D.L., Adams D.K., He J., Kalhor N., Zhang M., Xu T., Gao H., Reuben J.M., Qiao Y., Komaki R. (2017). Sequential Tracking of PD-L1 Expression and RAD50 Induction in Circulating Tumor and Stromal Cells of Lung Cancer Patients Undergoing Radiotherapy. Clin. Cancer Res..

[190] Kallergi G., Vetsika E.K., Aggouraki D., Lagoudaki E., Koutsopoulos A., Koinis F., Katsarlinos P., Trypaki M., Messaritakis I., Stournaras C. (2018). Evaluation of PD-L1/PD-1 on circulating tumor cells in patients with advanced non-small cell lung cancer. Ther. Adv. Med. Oncol..

[191] Wang Y., Kim T.H., Fouladdel S., Zhang Z., Soni P., Qin A., Zhao L., Azizi E., Lawrence T.S., Ramnath N. (2019). PD-L1 Expression in Circulating Tumor Cells Increases during Radio(chemo)therapy and Indicates Poor Prognosis in Non-small Cell Lung Cancer. Sci. Rep..

[192] Grizzi F., Castello A., Qehajaj D., Toschi L., Rossi S., Pistillo D., Paleari V., Veronesi G., Novellis P., Monterisi S. (2019). Independent expression of circulating and tissue levels of PD-L1: Correlation of clusters with tumor metabolism and outcome in patients with non-small cell lung cancer. Cancer Immunol. Immunother..

[193] Cheng Y., Wang T., Lv X., Li R., Yuan L., Shen J., Li Y., Yan T., Liu B., Wang L. (2020). Detection of PD-L1 Expression and Its Clinical Significance in Circulating Tumor Cells from Patients with Non-Small-Cell Lung Cancer. Cancer Manag. Res..

[194] Mazel M., Jacot W., Pantel K., Bartkowiak K., Topart D., Cayrefourcq L., Rossille D., Maudelonde T., Fest T., Alix-Panabieres C. (2015). Frequent expression of PD-L1 on circulating breast cancer cells. Mol. Oncol..

[195] Bergmann S., Coym A., Ott L., Soave A., Rink M., Janning M., Stoupiec M., Coith C., Peine S., von Amsberg G. (2020). Evaluation of PD-L1 expression on circulating tumor cells (CTCs) in patients with advanced urothelial carcinoma (UC). Oncoimmunology.

[196] Liu M., Wang R., Sun X., Liu Y., Wang Z., Yan J., Kong X., Liang S., Liu Q., Zhao T. (2020). Prognostic significance of PD-L1 expression on cell-surface vimentin-positive circulating tumor cells in gastric cancer patients. Mol. Oncol..

[197] Khattak M.A., Reid A., Freeman J., Pereira M., McEvoy A., Lo J., Frank M.H., Meniawy T., Didan A., Spencer I. (2020). PD-L1 Expression on Circulating Tumor Cells May Be Predictive of Response to Pembrolizumab in Advanced Melanoma: Results from a Pilot Study. Oncologist.

[198] Ilie M., Szafer-Glusman E., Hofman V., Chamorey E., Lalvee S., Selva E., Leroy S., Marquette C.H., Kowanetz M., Hedge P. (2018). Detection of PD-L1 in circulating tumor cells and white blood cells from patients with advanced non-small-cell lung cancer. Ann. Oncol..

[199] Wendel M., Bazhenova L., Boshuizen R., Kolatkar A., Honnatti M., Cho E.H., Marrinucci D., Sandhu A., Perricone A., Thistlethwaite P. (2012). Fluid biopsy for circulating tumor cell identification in patients with early-and late-stage non-small cell lung cancer: A glimpse into lung cancer biology. Phys. Biol.

[200] Liu H.E., Triboulet M., Zia A., Vuppalapaty M., Kidess-Sigal E., Coller J., Natu V.S., Shokoohi V., Che J., Renier C. (2017). Workflow optimization of genome amplification and targeted panel sequencing for CTC mutation detection. NPJ Genome Med..

[201] Picelli S., Faridani O.R., Bjorklund A.K., Winberg G., Sagasser S., Sandberg R. (2014). Full-length RNA-seq from single cells using Smart-seq2. Nat. Protoc..

[202] Sasagawa Y., Nikaido I., Hayashi T., Danno H., Uno K.D., Imai T., Ueda H.R. (2013). Quartz-Seq: A highly reproducible and sensitive single-cell RNA sequencing method, reveals non-genetic gene expression heterogeneity. Genome Biol..

[203] Hochegerner H., Lonnerberg P., Hodge R., Mikes J., Heskol A., Hubschle H., Lin P., Picelli S., Manno G.L., Ratz M. (2017). STRT-seq-2i: Dual-index 5’ single cell and nucleus RNA-seq on an addressable microwell array. Sci. Rep..

[204] Wang Z., Gerstein M., Snyder M. (2009). RNA-Seq: A revolutionary tool for transcriptomics. Nat. Rev. Genet..

[205] Sho S., Court C.M., Winograd P., Lee S., Hou S., Graeber T.G., Tseng H.R., Tomlinson J.S. (2017). Precision oncology using a limited number of cells: Optimization of whole genome amplification products for sequencing applications. BMC Cancer.

[206] Wang Q., Zhao L., Han L., Tuo X., Ma S., Wang Y., Feng X., Liang D., Sun C., Wang Q. (2019). The Discordance of Gene Mutations between Circulating Tumor Cells and Primary/Metastatic Tumor. Mol. Ther Oncolytics.

[207] Gulbahce N., Magbanua M.J.M., Chin R., Agarwal M.R., Luo X., Liu J., Hayden D.M., Mao Q., Ciotlos S., Li Z. (2017). Quantitative Whole Genome Sequencing of Circulating Tumor Cells Enables Personalized Combination Therapy of Metastatic Cancer. Cancer Res..

[208] De Luca F., Rotunno G., Salvianti F., Galardi F., Pestrin M., Gabellini S., Simi L., Mancini I., Vannucchi A.M., Pazzagli M. (2016). Mutational analysis of single circulating tumor cells by next generation sequencing in metastatic breast cancer. Oncotarget.

[209] Ni X., Zhuo M., Su Z., Duan J., Gao Y., Wang Z., Zong C., Bai H., Chapman A.R., Zhao J. (2013). Reproducible copy number variation patterns among single circulating tumor cells of lung cancer patients. Proc. Natl. Acad. Sci. USA.

[210] Scher H.I., Graf R.P., Schreiber N.A., McLaughlin B., Jendrisak A., Wang Y., Lee J., Greene S., Krupa R., Lu D. (2017). Phenotypic Heterogeneity of Circulating Tumor Cells Informs Clinical Decisions between AR Signaling Inhibitors and Taxanes in Metastatic Prostate Cancer. Cancer Res..

[211] D’Oronzo S., Lovero D., Palmirotta R., Stucci L.S., Tucci M., Felici C., Cascardi E., Giardina C., Cafforio P., Silvestris F. (2019). Dissection of major cancer gene variants in subsets of circulating tumor cells in advanced breast cancer. Sci. Rep..

[212] Oulhen M., Pawlikowska P., Tayoun T., Garonzi M., Buson G., Forcato C., Manaresi N., Aberlenc A., Mezquita L., Lecluse Y. (2021). Circulating tumor cell copy number heterogeneity in ALK-rearranged non-small-cell lung cancer resistant to ALK inhibitors. NPJ Precis. Oncol..

[213] Gracia D.F., Nteliopoulos G., Hastings R., Rushton A., Page K., Martinson L.J., Gray M., Guttery D.S., Ferrarini A., Manaresi N. (2021). Genomic copy number profiling of single CTCs reveals clonal evolution in metastatic breast cancer and identifies actionable targets for informing treatment decisions. AACR Cancer Res..

[214] Chang Y., Wang Y., Li B., Lu X., Wang R., Li H., Yan B., Gu A., Wang W., Huang A. (2021). Whole-Exome Sequencing on Circulating Tumor Cells Explores Platinum-Drug Resistance Mutations in Advanced Non-small Cell Lung Cancer. Front. Genet..

[215] Rossi E., Zamarchi R. (2019). Single-Cell analysis of circulating tumor cells: How far have we come in the -omics era?. Front. Genet..

[216] Hofman P. (2021). Next Generation sequencing with liquid biopsies from treatment naïve non small cell lung carcinoma patients. Cancers.

[217] Gawad C., Koh W., Quake S.R. (2016). Single-cell genome sequencing: Current state of the science. Nat. Rev. Genet..

[218] Lu S., Chang C.J., Guan Y., Szafer-Glusman E., Punnoose E., Do A., Suttmann B., Gagnon R., Rodriguez A., Landers M. (2020). Genomic Analysis of Circulating Tumor Cells at the Single-Cell Level. J. Mol. Diagn..

[219] Wu Y., Niu Y., Gao W., Shen X. (2021). Morphology classification of circulating tumor cells could be a predictor of recurrent disease in patients with non-small cell lung cancer after surgery. J. Clin. Oncol..

[220] de Bono J.S., Pantel K., Efstathiou E., Sternberg C.N., Castellano Gauna D., Fizazi K., Tomabl B., Wulfing C., Schonhoft J.D., Tubbs A. (2021). 614P Circulating tumor cell morphologic sub-types present prior to treatment in the CARD trial identify therapy resistance. Ann. Oncol..

[221] (2021). 20 Years of Precision Medicine in Oncology.

[222] Ignatiadis M., Sledge G.W., Jeffrey S.S. (2021). Liquid biopsy enters the clinic-implementaion issues and future challenges. Nat. Rev. Clin. Oncol..

[223] Hamza B., Miller A.B., Meier L., Stockslager M., Ng S.R., King E.M., Lin L., DeGouveia K.L., Mulugeta N., Calistri N.L. (2021). Measuring kinetics and metastatic propensity of CTCs by blood exchange between mice. Nat. Commun..

[224] Scherag F.D., Niestroj-Pahl R., Krusekopf S., Lucke K., Brandstetter T., Ruhe J. (2017). Highly Selective Capture Surfaces on Medical Wires for Fishing Tumor Cells in Whole Blood. Anal. Chem..

[225] Jaeger B.A.S., Neugebauer J., Andergassen U., Melcher C., Schochter F., Mouarrawy D., Ziemendorff G., Clemens M., Abel E.V., Heinrich G. (2017). The HER2 phenotype of circulating tumor cells in HER2-positive early breast cancer: A translational research project of a prospective randomized phase III trial. PLoS ONE.

[226] Ignatiadis M., Rothe F., Chaboteaux C., Durbecq V., Rouas G., Criscitiello C., Metallo J., Kheddoumi N., Singhal S.K., Michiels S. (2011). HER2-positive circulating tumor cells in breast cancer. PLoS ONE.

[227] Cao S., Li Y., Li J., Li C.F., Zhang W., Yang Z.Q., Meng S.D. (2010). Quantitative determination of HER2 expression by confocal microscopy assay in CTCs of breast cancer. Oncol. Rep..

[228] Romero D. (2016). Breast cancer: CTC heterogeneity is dynamic. Nat. Rev. Clin. Oncol..

[229] Keller L., Pantel K. (2019). Unravelling tumour heterogeneity by single-cell profiling of circulating tumour cells. Nat. Rev. Cancer.

[230] de Wit S., Manicone M., Rossi E., Lampignano R., Yang L., Zill B., Rengel-Puertas A., Ouhlen M., Crespo M., Berghuis A.M.S. (2018). EpCAM(high) and EpCAM(low) circulating tumor cells in metastatic prostate and breast cancer patients. Oncotarget.

[231] de Wit S., van Dalum G., Terstappen L.W. (2014). Detection of circulating tumor cells. Scientifica.

[232] Le Du F., Fujii T., Kida K., Davis D.W., Park M., Liu D.D., Wu W., Chavez-MacGregor M., Barcenas C.H., Valero V. (2020). EpCAM-independent isolation of circulating tumor cells with epithelial-to-mesenchymal transition and cancer stem cell phenotypes using ApoStream(R) in patients with breast cancer treated with primary systemic therapy. PLoS ONE.

[233] Raimondi C., Nicolazzo C., Gradilone A. (2015). Circulating tumor cells isolation: The “post-EpCAM era”. Chin. J. Cancer Res..

[234] Konigsberg R., Obermayr E., Bises G., Pfeiler G., Gneist M., Wrba F., de Santis M., Zeillinger R., Hudec M., Dittrich C. (2011). Detection of EpCAM positive and negative circulating tumor cells in metastatic breast cancer patients. Acta Oncol..

[235] Wu S., Liu S., Liu Z., Huang J., Pu X., Li J., Yang D., Deng H., Yang N., Xu J. (2015). Classification of circulating tumor cells by epithelial-mesenchymal transition markers. PLoS ONE.

[236] Papadaki M.A., Kallergi G., Zafeiriou Z., Manouras L., Theodoropoulos P.A., Mavroudis D., Georgoulias V., Agelaki S. (2014). Co-expression of putative stemness and epithelial-to-mesenchymal transition markers on single circulating tumour cells from patients with early and metastatic breast cancer. BMC Cancer.

[237] Qi L.N., Xiang B.D., Wu F.X., Ye J.Z., Zhong J.H., Wang Y.Y., Chen Y.Y., Chen Z.S., Ma L., Chen J. (2018). Circulating Tumor Cells Undergoing EMT Provide a Metric for Diagnosis and Prognosis of Patients with Hepatocellular Carcinoma. Cancer Res..

[238] Chen Y., Li S., Li W., Yang R., Zhang X., Ye Y., Yu J., Ye L., Tang W. (2019). Circulating tumor cells undergoing EMT are poorly correlated with clinical stages or predictive of recurrence in hepatocellular carcinoma. Sci. Rep..

[239] Murlidhar V., Rivera-Baez L., Nagrath S. (2016). Affinity versus Label-Free isolation of Circulating tumor cells: Who Wins?. Small.

[240] Wang Y., Guo L., Feng L., Zhang W., Xiao T., Di X., Chen G., Zhang K. (2018). Single nucleotide variant profiles of viable single circulating tumour cells reveal CTC behaviours in breast cancer. Oncol. Rep..

[241] Pestrin M., Salvianti F., Galardi F., De Luca F., Turner N., Malorni L., Pazzagli M., Di Leo A., Pinzani P. (2015). Heterogeneity of PIK3CA mutational status at the single cell level in circulating tumor cells from metastatic breast cancer patients. Mol. Oncol..

[242] Heitzer E., Auer M., Gasch C., Pichler M., Ulz P., Hoffmann E.M., Lax S., Waldispuehl-Geigl J., Mauermann O., Lackner C. (2013). Complex tumor genomes inferred from single circulating tumor cells by array-CGH and next-generation sequencing. Cancer Res..

[243] Carter L., Rothwell D.G., Mesquita B., Smowton C., Leong H.S., Fernandez-Gutierrez F., Li Y., Burt D.J., Antonello J., Morrow C.J. (2017). Molecular analysis of circulating tumor cells identifies distinct copy-number profiles in patients with chemosensitive and chemorefractory small-cell lung cancer. Nat. Med..

[244] Kwan T.T., Bardia A., Spring L.M., Giobbie-Hurder A., Kalinich M., Dubash T., Sundaresan T., Hong X., LiCausi J.A., Ho U. (2018). A Digital RNA Signature of Circulating Tumor Cells Predicting Early Therapeutic Response in Localized and Metastatic Breast Cancer. Cancer Discov..

[245] Miyamoto D.T., Lee R.J., Kalinich M., LiCausi J.A., Zheng Y., Chen T., Milner J.D., Emmons E., Ho U., Broderick K. (2018). An RNA-Based Digital Circulating Tumor Cell Signature Is Predictive of Drug Response and Early Dissemination in Prostate Cancer. Cancer Discov..

[246] Hu P., Zhang W., Xin H., Deng G. (2016). Single Cell Isolation and Analysis. Front. Cell Dev. Biol.

[247] Di Trapani M., Manaresi N., Medoro G. (2018). DEPArray system: An automatic image-based sorter for isolation of pure circulating tumor cells. Cytometry A.

[248] Nelep C., Eberhardt J. (2018). Automated rare single cell picking with the ALS cellcelector. Cytom. A.

[249] Swennenhuis J.F., Tibbe A.G., Stevens M., Katika M.R., van Dalum J., Tong H.D., van Rijn C.J., Terstappen L.W. (2015). Self-seeding microwell chip for the isolation and characterization of single cells. Lab Chip.

[250] Khoo B.L., Lee S.C., Kumar P., Tan T.Z., Warkiani M.E., Ow S.G., Nandi S., Lim C.T., Thiery J.P. (2015). Short-term expansion of breast circulating cancer cells predicts response to anti-cancer therapy. Oncotarget.

[251] Balakrishnan A., Koppaka D., Anand A., Deb B., Grenci G., Viasnoff V., Thompson E.W., Gowda H., Bhat R., Rangarajan A. (2019). Circulating Tumor Cell cluster phenotype allows monitoring response to treatment and predicts survival. Sci. Rep..

[252] Chen X., Gole J., Gore A., He Q., Lu M., Min J., Yuan Z., Yang X., Jiang Y., Zhang T. (2020). Noninvasive early detection of cancer four years before conventional diagnosis using a blood-test. Nat. Commun..

[253] Nabet B.Y., Esfashin M.S., Moding E.J., Hamilton E.G., Chabon J.J., Rizvi H., Steen C.B., Chaudhuri A.A., Liu C.L., Hui A.B. (2020). Noninvasive early identification of therapeutic benefit from immune checkpoin inhibition. Cell.

